# Defining endogenous TACC3–chTOG–clathrin–GTSE1 interactions at the mitotic spindle using induced relocalization

**DOI:** 10.1242/jcs.255794

**Published:** 2021-02-01

**Authors:** Ellis L. Ryan, James Shelford, Teresa Massam-Wu, Richard Bayliss, Stephen J. Royle

**Affiliations:** 1 Centre for Mechanochemical Cell Biology, Warwick Medical School, Gibbet Hill Road, Coventry CV4 7AL, UK; 2 School of Molecular and Cellular Biology, Astbury Centre for Structural Biology, Faculty of Biological Sciences, University of Leeds, Leeds LS2 9JT, UK

**Keywords:** GTSE1, TACC3, Clathrin, Knocksideways, Mitosis, Mitotic spindle

## Abstract

A multiprotein complex containing TACC3, clathrin and other proteins has been implicated in mitotic spindle stability. To disrupt this complex in an anti-cancer context, we need to understand its composition and how it interacts with microtubules. Induced relocalization of proteins in cells is a powerful way to analyze protein–protein interactions and, additionally, monitor where and when these interactions occur. We used CRISPR/Cas9 gene editing to add tandem FKBP–GFP tags to each complex member. The relocalization of endogenous tagged protein from the mitotic spindle to mitochondria and assessment of the effect on other proteins allowed us to establish that TACC3 and clathrin are core complex members and that chTOG (also known as CKAP5) and GTSE1 are ancillary to the complex, binding respectively to TACC3 and clathrin, but not each other. We also show that PIK3C2A, a clathrin-binding protein that was proposed to stabilize the TACC3–chTOG–clathrin–GTSE1 complex during mitosis, is not a member of the complex. This work establishes that targeting the TACC3–clathrin interface or their microtubule-binding sites are the two strategies most likely to disrupt spindle stability mediated by this multiprotein complex.

## Abstract (precis)

**Summary:** The composition of the mitotic TACC3–chTOG–clathrin–GTSE1 complex and its interaction with microtubules is determined using induced relocalization of endogenous proteins from the spindle to mitochondria.

## INTRODUCTION

During mitosis, chromosomes are segregated with high precision to generate two genetically identical daughter cells. This segregation is driven by the mitotic spindle, a bipolar microtubule array with associated motor and non-motor proteins (Manning and Compton, 2008). One non-motor protein complex that binds spindle microtubules contains TACC3, chTOG (also known as CKAP5) and clathrin (Fu et al., 2010; Hubner et al., 2010; Lin et al., 2010; Booth et al., 2011). This complex is important for stabilizing the bundles of microtubules that attach to kinetochores (kinetochore-fibers, k-fibers) by physically crosslinking them (Booth et al., 2011; Hepler et al., 1970; Nixon et al., 2015, 2017). Uncovering the molecular details of how proteins of this complex bind to one another and to microtubules is important to understand how mitotic spindles are stabilized and how we can target spindle stability in an anti-cancer context.

Mitotic phosphorylation of TACC3 on serine 558 by Aurora kinase A (referred to here as Aurora A) controls the interaction between clathrin and TACC3 (Booth et al., 2011; Cheeseman et al., 2011, 2013; Hood et al., 2013; Burgess et al., 2018). This interaction brings together the N-terminal domain of clathrin heavy chain and the TACC domain of TACC3 to make the microtubule-binding surface (Hood et al., 2013). Despite having a microtubule-lattice binding domain, chTOG is not needed for the complex to bind microtubules and interacts with the TACC3–clathrin complex via its TOG6 domain, binding to a stutter in the TACC domain of TACC3 (Booth et al., 2011; Hood et al., 2013; Gutiérrez-Caballero et al., 2015).

Despite this detail, the exact composition of the complex on kinetochore microtubules is uncertain. Besides TACC3, clathrin and chTOG, two further proteins, GTSE1 and phosphatidylinositol 4-phosphate 3-kinase C2 domain-containing subunit α (PI3K-C2α, also known as PIK3C2A) have been proposed to be members. Both were originally identified as binding partners for mitotic TACC3–clathrin (Hubner et al., 2010). Biochemical evidence convincingly shows that GTSE1 binds the N-terminal domain of clathrin heavy chain and that this interaction localizes GTSE1 to spindle microtubules (Rondelet et al., 2020). Like chTOG, GTSE1 has the capacity to bind microtubules, but it appears to use TACC3–clathrin to bind the spindle (Monte et al., 2000; Scolz et al., 2012; Bendre et al., 2016). By contrast, PIK3C2A is a component of clathrin-coated vesicles where it acts as a lipid kinase (Gaidarov et al., 2001). It was recently proposed to act as a scaffolding protein in the TACC3–chTOG–clathrin complex by binding to both TACC3 and clathrin (Gulluni et al., 2017). PIK3C2A and GTSE1 bind to the same sites on the N-terminal domain of clathrin heavy chain (Gaidarov et al., 2001; Rondelet et al., 2020), and although clathrin has the capacity to bind multiple proteins (Smith et al., 2017; Willox and Royle, 2012), this raises the question of whether the binding of PIK3C2A and GTSE1 to TACC3–clathrin at the spindle is mutually exclusive.

Dissecting this multiprotein complex is further complicated by each putative member being able to form subcomplexes that have different subcellular localizations (Gutiérrez-Caballero et al., 2015). TACC3–chTOG (without clathrin) localizes to the plus ends of microtubules (Nwagbara et al., 2014; Gutiérrez-Caballero et al., 2015). Similarly, GTSE1 binds plus ends and can also stabilize astral microtubules of the mitotic spindle by inhibiting the microtubule depolymerase MCAK (also known as KIF2C; Scolz et al., 2012; Bendre et al., 2016; Tipton et al., 2017). PIK3C2A and clathrin are found in clathrin-coated vesicles away from the mitotic spindle (Gaidarov et al., 2001). Biochemical approaches do not have the capacity to discriminate these subcomplexes from the multiprotein complex on k-fibers. Therefore, subcellular investigation of protein interactions are required to answer this question.

Knocksideways is a method to acutely and inducibly relocalize a protein to mitochondria in order to inactivate that protein (Robinson et al., 2010). In the original method, the target protein is depleted by RNAi, and an FKBP-tagged version is expressed alongside MitoTrap (an FRB domain targeted to mitochondria); relocalization is achieved by the addition of rapamycin. This method has many advantages over slow inactivation methods such as RNAi-mediated knockdown or gene disruption (knockout) approaches (Royle, 2013). We have previously used knocksideways in mitotic cells to investigate protein–protein interactions, because any proteins that are in a complex with the target protein also become mislocalized to the mitochondria (Cheeseman et al., 2013; Hood et al., 2013). This approach has the added advantage that the subcellular location of proteins can also be tracked during the experiment, and that it can be done at specific times, allowing us to pinpoint where and when interactions occur.

In this study, we applied a knocksideways approach to investigate how proteins of the TACC3–chTOG–clathrin–GTSE1 complex bind to one another and to microtubules of the mitotic spindle. Instead of overexpression and RNAi, we sought to tag each target protein with FKBP and GFP at their endogenous locus using CRISPR/Cas9-mediated gene editing. This strategy allowed us to study these subcellular interactions at the endogenous level for the first time. The cell lines we have created are a multi-purpose ‘toolkit’ for studying microtubule-crosslinking proteins by live-cell imaging, biochemistry or electron microscopy (Clarke and Royle, 2018).

## RESULTS

### Generation and validation of clathrin, TACC3, chTOG and GTSE1 knock-in HeLa cell lines

**Fig. 1.**
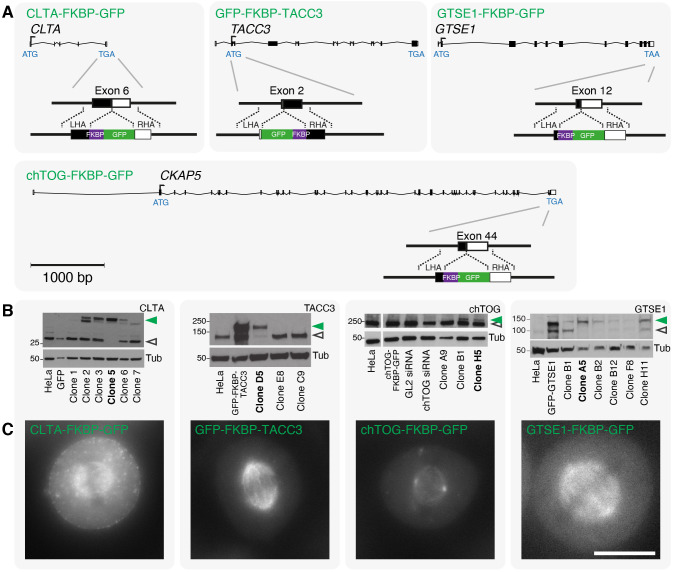
**Generation of knock-in HeLa cell lines using gene editing.** (A) Strategy to tag clathrin (CLTA), TACC3, chTOG (CKAP5) and GTSE1 with FKBP and GFP at their endogenous loci. Cas9n D10A nickase was used to target the indicated site, and a repair template with FKBP–GFP or GFP–FKBP tag flanked by left and right homology arms (LHA and RHA, respectively) was used as indicated. GFP-positive cells were individually sorted by FACS and validated using a combination of western blotting, imaging and DNA sequencing (not shown). (B) Western blotting showed negative clones and positive clones that were either homozygous (single band, shifted by ∼30 kDa) or heterozygous (two bands, one at expected size and the other shifted by ∼30 kDa) knock-in cell lines. The tagged and untagged proteins are denoted by filled green and open gray arrowheads, respectively. Clones used in this work are highlighted in bold. Molecular mass markers are shown in kDa for each blot. Control lanes show parental HeLa (HeLa) or parental HeLa overexpressing the indicated protein. HeLa cells may have more than two alleles of the targeted gene. We use the term homozygous to indicate editing of all alleles and heterozygous to indicate that at least one allele was edited and that an unedited allele remained. PCR and DNA sequencing confirmed that: CLTA–FKBP–GFP (clone 5) is homozygous, GFP–FKBP–TACC3 (clone D5) is homozygous, chTOG–FKBP–GFP (clone H5) is heterozygous and GTSE1–FKBP–GFP (clone A5) is homozygous. (C) Micrographs showing GFP fluorescence of each tagged cell line indicating the correct localization of each tagged protein. Scale bar: 10 µm.

Our first goal was to tag four proteins with FKBP and GFP at their endogenous loci using CRISPR/Cas9 gene editing. Clathrin (targeting clathrin light chain A, LCa, also known as CLTA), TACC3, chTOG (CKAP5) and the clathrin-interacting protein GTSE1 were edited in HeLa cells so that they had a GFP–FKBP tag at their N-terminus or an FKBP–GFP tag at their C-terminus (Fig. 1A). The dual FKBP and GFP tag allows direct visualization of the protein as well as its spatial manipulation using knocksideways (Cheeseman et al., 2013; Robinson et al., 2010). Following editing, GFP-positive cells were isolated by FACS and were validated using a combination of PCR, sequencing, western blotting and fluorescence microscopy (Fig. 1B,C; Figs S1, S2). These validation steps yielded a cell line for each protein that could be used for all future analyses. Homozygous knock-in was achieved for CLTA–FKBP–GFP, GFP–FKBP–TACC3 and GTSE1–FKBP–GFP. Despite multiple attempts to generate a homozygous knock-in for chTOG–FKBP–GFP, we only recovered heterozygous lines (more than twenty heterozygous clones in three separate attempts). Although there is a report of homozygous knock-in of chTOG–FKBP–GFP in HCT116 cells (Cherry et al., 2019), we assume that homozygous knock-in of chTOG–FKBP–GFP in HeLa cells is lethal.

The localization of tagged proteins in all cell lines was normal. In mitotic cells, clathrin was located on the spindle, in the cytoplasm and at coated pits; TACC3 was located exclusively on the spindle; chTOG was located on the spindle but was more pronounced on the centrosomes and kinetochores; and GTSE1 was localized throughout the spindle and the cytoplasm (Fig. 1C; Fig. S2A,C), consistent with previous observations (Gergely et al., 2000, 2003; Royle et al., 2005; Foraker et al., 2012; Bendre et al., 2016; Herman et al., 2020). Overexpression of TACC3 can result in the formation of aggregates (Gergely et al., 2000; Hood et al., 2013), which have recently been described as liquid-like phase-separated structures (So et al., 2019). We note that at endogenous levels in HeLa cells, GFP–FKBP–TACC3 did not form these structures (Fig. 1C; Fig. S2A). GTSE1–FKBP–GFP could be seen tracking microtubule plus ends in interphase, as previously reported (Scolz et al., 2012), but also during all stages of mitosis (Fig. S2D).

As a further validation step, we assessed mitotic timings in each knock-in cell line and found progression to be comparable to that of their respective parental HeLa cells. These observations indicate that the addition of an FKBP and GFP tag did not affect the mitotic function of clathrin, TACC3, chTOG or GTSE1, and that clonal selection did not adversely affect mitosis in the four cell lines (Fig. S3). In summary, the generation and validation of these four knock-in cell lines represents a toolkit that can be used to study clathrin, TACC3, chTOG and GTSE1 at endogenous levels (see Table S1).

### Knocksideways of endogenous proteins in knock-in cell lines

**Fig. 2.**
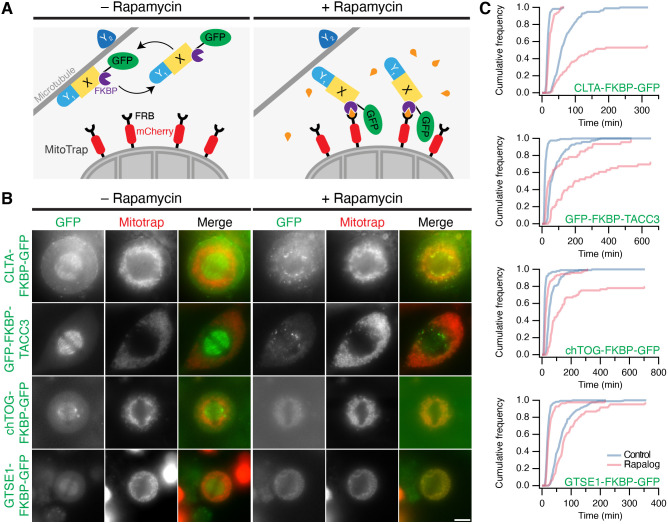
**Generation of knock-in HeLa cell lines using gene editing.** (A) Schematic diagram of knocksideways in gene edited cells. A microtubule-binding protein X is fused to FKBP and GFP. MitoTrap, an FRB domain targeted to mitochondria, tagged with mCherry, is transiently expressed. Addition of rapamycin causes the relocalization of proteins to the mitochondria (Robinson et al., 2010). This strategy can also be used to assess whether another protein Y, co-reroutes with X to the mitochondria. Y_1_ co-reroutes with X, indicating that they form a complex, whereas Y_2_ does not. (B) Live-cell imaging of knocksideways of gene-edited cell lines. The indicated tagged cell lines expressing mCherry-tagged MitoTrap were imaged on a widefield microscope. Stills from a movie where metaphase cells were treated with rapamycin (200 nM) are shown. The post-rapamycin images (+ Rapamycin) are 10–15 min after treatment. Scale bar: 10 µm. (C) Mitotic progression following knocksideways. Cumulative histograms of timings from nuclear envelope breakdown (NEB) to metaphase (short duration plots) and NEB to anaphase (long duration plots). Gene-edited cells expressing mCherry–MitoTrap(T2098L) were pre-treated with 1 µM rapalog as indicated. All imaging experiments were repeated three times. Number of cells analyzed (control and rapalog, respectively): CLTA–FKBP–GFP, 115 and 66; GFP–FKBP–TACC3, 122 and 46; chTOG–FKBP–GFP, 104 and 73; GTSE1–FKBP–GFP, 176 and 84.

We next performed knocksideways experiments to assess the functionality of the FKBP tag that was introduced (Fig. 2A). Each cell line, expressing mCherry–MitoTrap, was imaged live during the application of 200 nM rapamycin. At metaphase, CLTA–FKBP–GFP, GFP–FKBP–TACC3, chTOG–FKBP–GFP and GTSE1–FKBP–GFP were all removed from the spindle and relocalized to the mitochondria by rapamycin addition (Fig. 2B). The timecourse of relocalization was variable but was complete by 10 min (Movies 1–4). The efficiency of relocalization in all four cell lines was 100% (clathrin, 28/28; TACC3, 26/26; chTOG, 22/22; GTSE1, 20/20).

To test whether relocating the tagged protein from the spindle to the mitochondria was sufficient to induce a mitotic phenotype, we analyzed progression through mitosis. Each knock-in cell line, transiently expressing mCherry–MitoTrap(T2098L), a rapalog-sensitive MitoTrap, was imaged overnight by light microscopy following application of 1 µM rapalog AP21967 or control. Relocalization of endogenous clathrin (CLTA–FKBP–GFP) caused a prolonged mitosis (median nuclear envelope breakdown-to-anaphase timing of 73*.*5 min versus 57*.*75 min in control conditions), with only 55% of cells exiting metaphase during the movie compared with 100% of control (Fig. 2C). Similarly, TACC3 relocalization prolonged the time to reach anaphase by 2.2-fold compared to the time taken by the control, with delays in reaching metaphase and reaching anaphase, and 72% of cells exiting metaphase. Relocalization of chTOG (chTOG–FKBP–GFP) also caused a delay of 1.5-fold, with 80% of cells entering anaphase. This phenotype was more mild than that following clathrin or TACC3 relocalization, although we note that some chTOG is likely to remain on the spindle due to the heterozygosity of this cell line. Finally, relocalization of GTSE1–FKBP–GFP had a smaller effect on mitotic progression, with 96% of cells exiting metaphase and a 1.2-fold delay in the timing from nuclear envelope breakdown to anaphase (Fig. 2C). These experiments show that relocalization of each protein is possible in live cells using knocksideways and that functional mitotic consequences of this mislocalization can be observed.

### Defining mitotic clathrin, TACC3, chTOG and GTSE1 interactions using knocksideways of endogenously tagged proteins

**Fig. 3.**
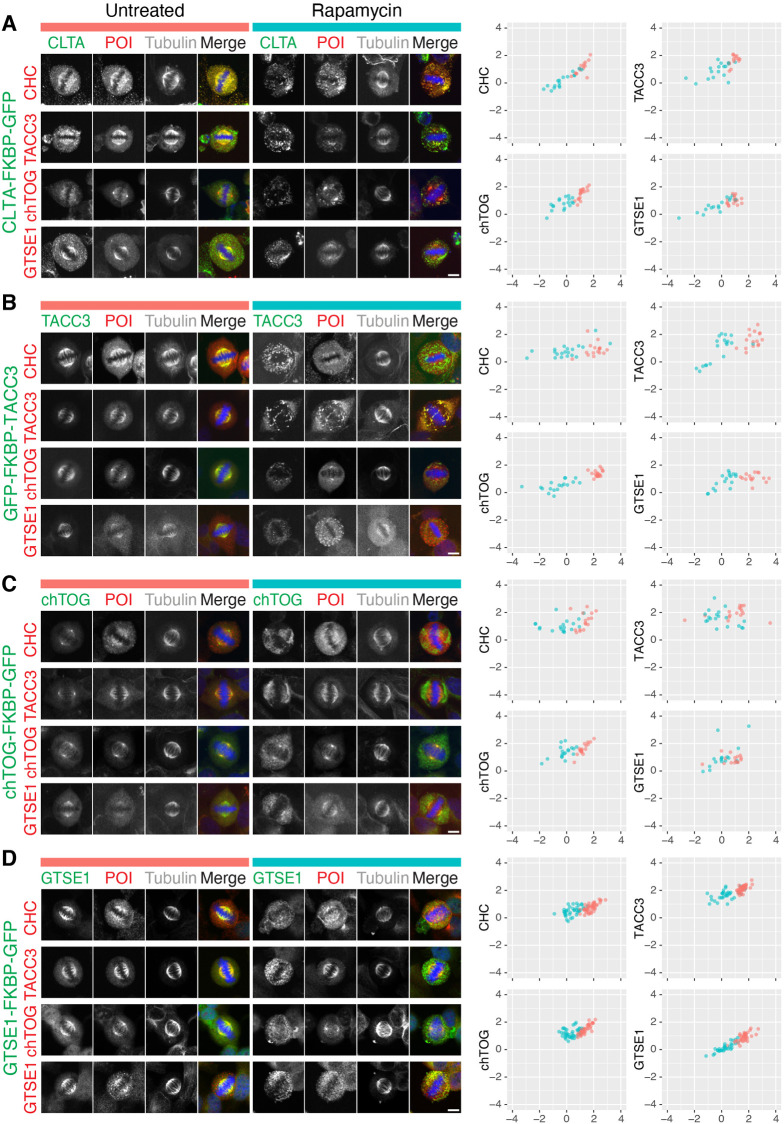
**Co-rerouting of endogenous complex members following knocksideways in knock-in cell lines.** (A–D) Knocksideways experiments using each knock-in cell line expressing dark MitoTrap. (A) CLTA–FKBP–GFP cells. (B) GFP–FKBP–TACC3 cells. (C) chTOG–FKBP–GFP cells. (D) GTSE1–FKBP–GFP cells. Representative confocal micrographs of cells that were either untreated or treated with rapamycin (200 nM) for 30 min, fixed and stained for tubulin and either CHC, TACC3, chTOG or GTSE1 (protein of interest, POI; red). Scale bars: 10 µm. Right, quantification of images. Spindle localization of the target protein (*x*-axis) and the POI (*y*-axis) in control (red) and knocksideways (turquoise) cells. Spindle localization is the ratio of spindle to cytoplasmic fluorescence shown on a log_2_ scale (where a value of 1 is twice the amount of fluorescence signal in the spindle regions of interest as in the cytoplasmic regions of interest, and −1 indicates half the amount of fluorescence in the spindle regions of interest versus in the cytoplasmic regions of interest). Quantification of cells from three or more experiments is shown.

Acute manipulation of protein localization using knocksideways can be used to uncover interactions in living cells (Hood et al., 2013). To examine mitotic interactions between clathrin, TACC3, chTOG and GTSE1, we set out to relocalize each endogenous protein in mitotic knock-in cells and ask whether this manipulation affects the localization of the other proteins, detected by indirect immunofluorescence (Fig. 3). Relocalization of endogenous clathrin (CLTA–FKBP–GFP) caused the removal of TACC3, GTSE1 and chTOG from the spindle (Fig. 3A). On the other hand, relocalization of endogenous GFP–FKBP–TACC3 resulted in removal of chTOG but only small reduction in clathrin and GTSE1 (Fig. 3B). By contrast, relocalization of chTOG–FKBP–GFP had no effect on the spindle localization of the other three proteins (Fig. 3C). We also detected small changes in clathrin, TACC3 and chTOG localization following relocalization of GTSE1–FKBP–GFP (Fig. 3D). Although these experiments were designed to examine interactions between endogenous proteins, it is only possible to measure relocalization and removal in different populations of cells.

**Fig. 4.**
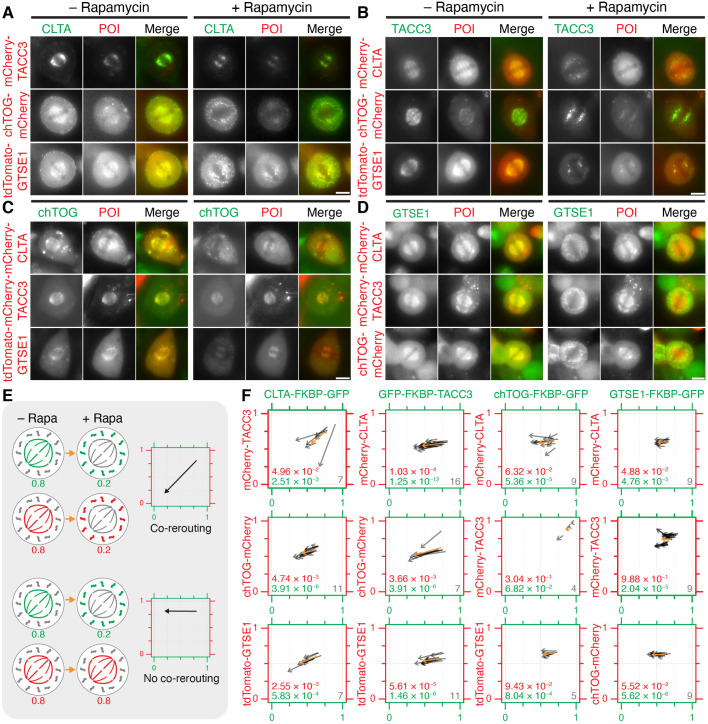
**Co-rerouting of complex members during live-cell knocksideways experiments in knock-in cell lines.** (A–F) Knocksideways experiments using each knock-in cell line expressing dark MitoTrap and one of the three other complex proteins tagged with a red fluorescent protein (mCherry–CLTA, mCherry–TACC3, chTOG–mCherry or tdTomato–GTSE1) as indicated. (A) CLTA–FKBP–GFP cells. (B) GFP–FKBP–TACC3 cells. (C) chTOG–FKBP–GFP cells. (D) GTSE1–FKBP–GFP cells. Still images are shown before (− Rapamycin) and 10 min after rapamycin (200 nM; + Rapamycin) treatment. In merge panels, GFP fluorescence is shown in green and protein of interest (POI) fluorescence is shown in red. Scale bars: 10 µm. (E) Explanation of ‘arrow plots’ to analyze co-rerouting. Arrows show the fraction of combined spindle and mitochondria fluorescence that is at the spindle (i.e. 1=completely spindle-localized, 0=mitochondria-localized) for green and red fluorescence channels, moving from pre- to post-rapamycin localization. Examples are shown of two mCherry-tagged proteins that do (top) or do not (bottom) co-reroute with an FKBP–GFP-tagged protein. (F) Arrow plots of live-cell knocksideways experiments. Gray arrows represent individual cells measured across three experimental repeats (*n* is shown bottom right), orange arrow indicates the mean. Bottom left of each plot, *P*-values from Student's paired *t*-tests to compare the effect of rapamycin on the two proteins in that condition.

We next sought to repeat these experiments using a single-cell live-imaging approach. To do this, the knock-in cell lines were transfected with dark MitoTrap and either mCherry–CLTA, mCherry–TACC3, chTOG–mCherry or tdTomato–GTSE1. Metaphase cells were imaged live as rapamycin was added (Fig. 4). Relocalization of endogenous clathrin caused the removal of mCherry–TACC3, chTOG–mCherry and tdTomato–GTSE1 from the spindle (Fig. 4A). Similarly, relocalization of endogenous TACC3 also caused the removal of the other three proteins from the spindle, but to a lesser extent than with clathrin relocalization (Fig. 4B). Again, relocalization of either chTOG or GTSE1 had no effect on the spindle localization of the other three proteins (Fig. 4C,D). A semi-automated analysis procedure was used to measure induced relocalization of both proteins (see Materials and Methods). All movement was from the mitotic spindle to the mitochondria, without significant loss to the cytoplasm, suggesting that the complex is either relocalized en masse or not. Two-dimensional arrow plots were therefore used to visualize the results of these experiments (Fig. 4E,F). As previously reported, mCherry–TACC3 expression distorted the localization of the complex prior to knocksideways (Booth et al., 2011; Nixon et al., 2015), enhancing the amount of clathrin, chTOG and GTSE1 on the spindle (Fig. 4F, note the rightward shift of the starting point in the arrow plots when mCherry–TACC3 was expressed). This likely reflects the importance of TACC3 in loading the complex onto the spindle (Hood et al., 2013). The expression of other partner proteins, mCherry–CLTA, chTOG–mCherry and tdTomato–GTSE1, had no effect on the localization of the knock-in protein.

The lack of removal of complex members after relocalization of chTOG–FKBP–GFP could be due to the heterozygosity of this knock-in cell line, since the untagged copy may prevent removal. In order to verify this result, we performed knocksideways using transient expression of chTOG–FKBP–GFP in unedited HeLa cells that were depleted of endogenous chTOG by RNAi. These experiments showed that clathrin, TACC3 and GTSE1 all remain in place following the relocalization of chTOG–GFP–FKBP to the mitochondria (Fig. S4).

The results of both knocksideways approaches are summarized in Table S2. Overall, the relocalization of either clathrin or TACC3 during metaphase results in removal of the entire TACC3–chTOG–clathrin–GTSE1 complex. The efficiency of this removal is higher with clathrin than TACC3, yet overexpression of TACC3 can load more complex members onto the spindle. Relocalization of either chTOG or GTSE1 has no effect on the rest of the complex, suggesting that these proteins are ancillary to TACC3–chTOG–clathrin–GTSE1, while TACC3 and clathrin are core members.

### Role of LIDL motifs in recruitment of GTSE1 to the TACC3–chTOG–clathrin complex

**Fig. 5.**
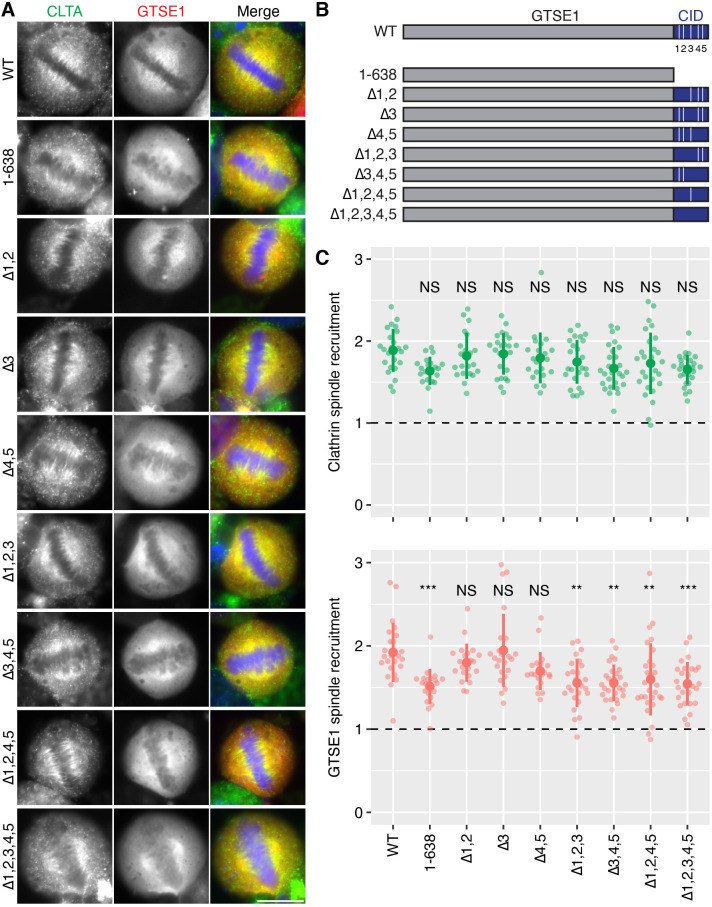
**Role of LIDL motifs in GTSE1 spindle localization.** (A) Representative widefield micrographs of GTSE1–mCherry constructs (red) in GTSE1-depleted CLTA–FKBP–GFP cells at metaphase. Cells expressing the indicated constructs, as described in B, were fixed and stained using DAPI (blue) and a GFP-boost antibody to enhance the signal of CLTA–FKBP–GFP (green). Scale bar: 10 µm. (B) Schematic diagram of full-length GTSE1 (WT, 1–720), truncated GTSE1 lacking the CID (1–638) and mutant forms. The five LIDL motifs (white) are numbered 1 to 5. Mutation of the corresponding motifs by replacement of each motif sequence with four alanine residues is denoted by Δ. (C) Quantification of the spindle localization of clathrin (top) and GTSE1 (bottom). Each dot represents a single cell, *n*=21–28 cells per construct over three separate experiments. The dashed horizontal line represents no enrichment on the spindle. The large dot and error bars show the mean±s.d., respectively. ANOVA with Tukey's post-hoc test was used to compare the means between each group. The *P*-value level is shown compared to WT: ***P<0*.*001; **P<0.01; NS, *P>*0.05.

In order to test if GTSE1 is an ancillary complex member, we sought to disrupt its interaction with clathrin and assess whether or not the spindle-binding of these two proteins was interdependent. To examine the effect on the mitotic localization of both proteins, mCherry-tagged GTSE1 constructs were expressed in GTSE1-depleted CLTA–FKBP–GFP cells (Fig. 5). GTSE1 has a previously mapped clathrin-interaction domain (CID; amino acids 639–720) containing five clathrin box-like motifs (LI[DQ][LF]; hereafter referred to as LIDL motifs), which was targeted for disruption (Wood et al., 2017; Rondelet et al., 2020). We found that deletion of the entire CID resulted in a reduction in GTSE1 on the spindle. Mutation of LIDL motifs 1 and 2, 3, or 4 and 5 to alanines did not result in reduction, but when mutated in combination resulted in a loss of GTSE1 that was similar to deletion of the CID. However, under all conditions the spindle localization of clathrin was unaffected. These findings were corroborated by a live-cell knocksideways approach (Fig. S5).

To test whether the reduction in GTSE1 spindle localization represented a block of recruitment, cells were treated with 0*.*3 µM MLN8237 to inhibit Aurora A activity and provide a reference for minimal recruitment (Hood et al., 2013; Booth et al., 2011). Spindle localization of both clathrin and wild-type GTSE1 (WT) was abolished by drug treatment (Fig. S6). Again, spindle localization of GTSE1 with LIDL motifs 1–5 mutated to alanine (GTSE1Δ1,2,3,4,5) was lower than that of WT in untreated cells, and was not reduced further by MLN8237 treatment (*P*=0.08). These data are consistent with the idea that GTSE1 is recruited to the spindle by clathrin via multiple LIDL motifs in GTSE1 (Rondelet et al., 2020). Moreover, they suggest that there is no interdependent spindle localization of clathrin–GTSE1 and that GTSE1 is an ancillary member of the complex.

**Fig. 6.**
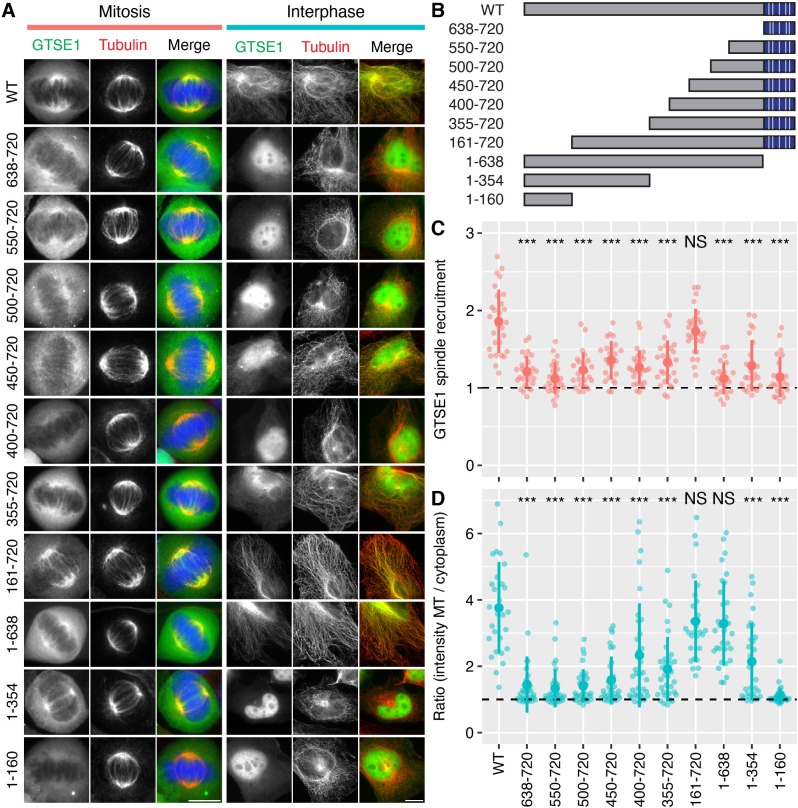
**Localization of GTSE1 fragments in interphase and mitosis.** (A) Representative widefield micrographs of GTSE1–FKBP–GFP constructs (green), as described in B, expressed in cells in mitosis or interphase. Cells were stained to show α-tubulin (red) and DNA (cells in mitosis only; DAPI, blue). Scale bars: 10 µm. (B) Schematic diagram of full-length GTSE1 (WT, 1–720) and fragments of GTSE1 used in this figure. The CID is shown in blue, with LIDL motifs indicated by white lines. Quantification of GTSE1 localization on mitotic spindles (C) or interphase microtubules (D). Each dot represents a single cell, *n*=23–28 cells per construct (mitosis) and *n*=27–33 cells per construct (interphase) pooled from three independent experiments. The dashed horizontal line represents no enrichment. The large dot and error bars show the mean±s.d., respectively. ANOVA with Tukey's post-hoc test was used to compare the means between each group. The *P*-value level is shown compared to WT: ***P<0.001; NS, *P>*0.05.

The ability to bind clathrin is necessary for GTSE1 to localize to the spindle, but is it sufficient? To address this question we examined the subcellular localization of a panel of GTSE1 fragments in mitosis and in interphase cells (Fig. 6). A GTSE1 fragment comprising the CID containing all five LIDL motifs was unable to bind the mitotic spindle. Progressively adding more N-terminal sequence to the CID eventually yielded a construct that bound the spindle (amino acids 161–720; Fig. 6A–C). This experiment demonstrated that the CID alone is not sufficient for spindle localization. Interphase microtubule binding was seen for the GTSE1 fragment 161–720 and to a lesser extent for 1–354, 335–720 and 400–720 (Fig. 6A,D). This suggests that the region 161–638 contains one or more regions that can bind microtubules and that these regions, together with the five LIDL motifs in the CID, are required for spindle localization.

### PIK3C2A is not a component of the TACC3–chTOG–clathrin–GTSE1 complex

**Fig. 7.**
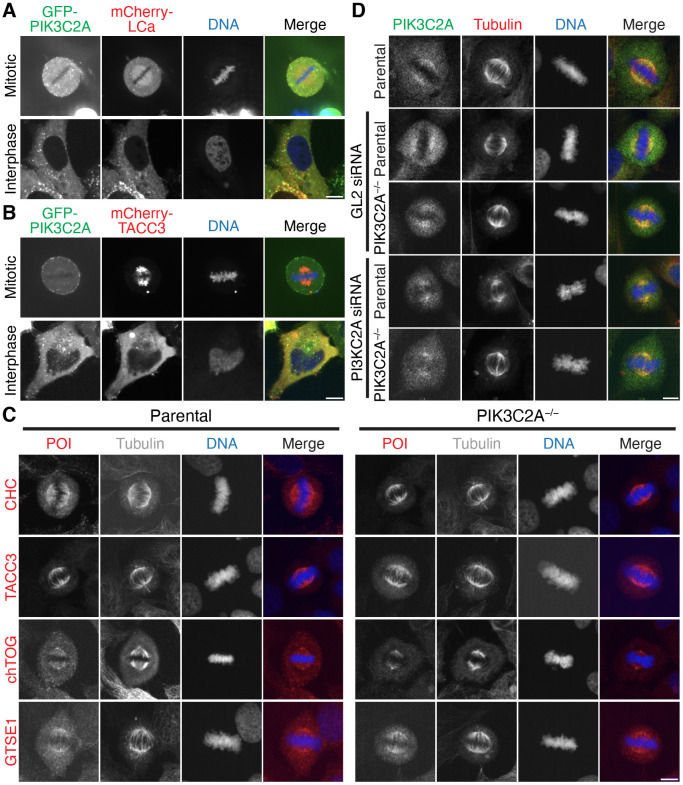
**PIK3C2A is not a component of the TACC3–chTOG–clathrin–GTSE1 complex.** (A,B) Representative confocal micrographs of mitotic and interphase HeLa cells expressing GFP–PIK3C2A and either (A) mCherry–CLTA (mCherry–LCa) or (B) mCherry–TACC3. (C) Representative widefield micrographs of parental HeLa and PIK3C2A-null (PIK3C2A^−/−^) cells stained for tubulin and either CHC, TACC3, chTOG or GTSE1 (red; protein of interest, POI). (D) Representative widefield micrographs of parental HeLa and PIK3C2A^−/−^ cells treated with GL2 (control) or PIK3C2A siRNA, stained with an anti-PIK3C2A antibody (Proteintech; green) and an anti-tubulin antibody (red). DNA was stained with DAPI (blue). Scale bars: 10 µm.

We next investigated whether or not PIK3C2A is a component of the TACC3–chTOG–clathrin–GTSE1 complex, since PIK3C2A has been proposed to bind TACC3 and clathrin, and therefore stabilize the complex (Gulluni et al., 2017). If PIK3C2A binds the complex, we would predict that it should also localize to the mitotic spindle. We imaged GFP–PIK3C2A in live cells and found no evidence for spindle localization (Fig. 7A). The construct localized to clathrin-coated pits, suggesting that the GFP tag had not interfered with its normal localization. We next overexpressed mCherry–TACC3 to concentrate the TACC3–chTOG–clathrin–GTSE1 complex on the spindle and maximize our chances of seeing any GFP–PIK3C2A signal on microtubules but, again, we saw no spindle localization of GFP–PIK3C2A (Fig. 7B).

To further explore any mitotic role for PIK3C2A, we generated a PIK3C2A-knockout cell line using CRISPR/Cas9. This generated a clone with a premature stop codon in both alleles, resulting in truncation after 87 and 72 residues for the two alleles, that we termed PIK3C2A null (Fig. S7C). It was previously shown that PIK3C2A knockout in primary mouse embryo fibroblasts (MEFs) altered their mitotic progression (Gulluni et al., 2017). We analyzed mitotic timings of our PIK3C2A-null cell line, compared to those of parental HeLa cells, and found no differences in mitotic timings (Fig. S7D).

If PIK3C2A was a scaffold protein for the TACC3–chTOG–clathrin–GTSE1 complex, we would expect some disruption of the spindle localization of clathrin, TACC3, chTOG or GTSE1 in the PIK3C2-null cells. However, immunostaining of parental HeLa and PIK3C2A-null cells with antibodies against clathrin heavy chain (CHC), TACC3, chTOG and GTSE1 revealed a similar distribution of all four complex members during mitosis (Fig. 7C). In the original paper, immunostaining of PIK3C2A at the mitotic spindle was shown (Gulluni et al., 2017).

We immunostained parental HeLa cells and the PIK3C2A-null cells with the same anti-PI3KC3A antibody used in the original report and found that there was a signal at the mitotic spindle, but that it was non-specific, because it was also detected in the PIK3C2A-null cells (Fig. 7D). We also used RNAi of PIK3C2A in parental and PIK3C2A-null cells to rule out the possibility that the antibody signal resulted from residual expression of PIK3C2A. Again, the spindle fluorescence remained after RNAi treatment, indicating that the antibody is non-specific for immunofluorescence. Taken together, our results suggest that PIK3C2A is not a component of the TACC3–chTOG–clathrin–GTSE1 complex.

## DISCUSSION

**Fig. 8.**
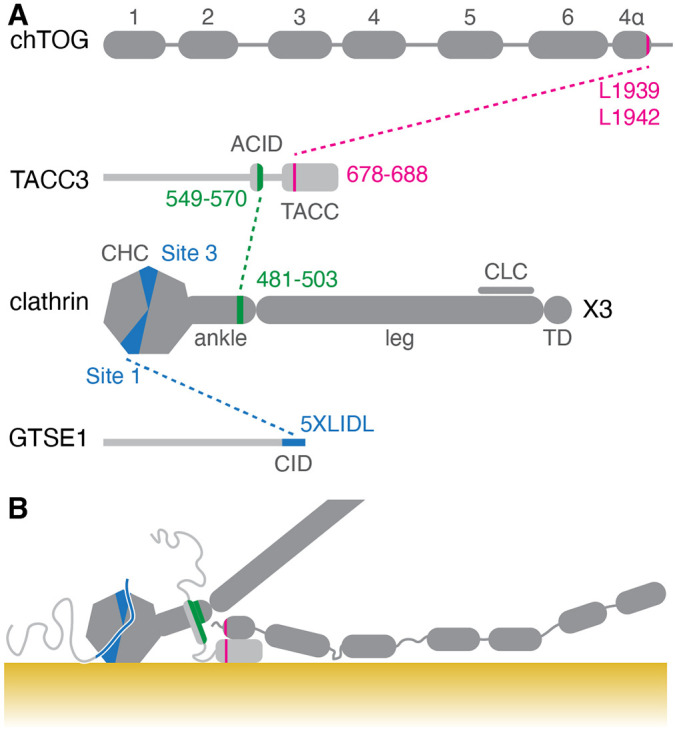
**Summary diagram of interactions between TACC3–chTOG–clathrin–GTSE1 complex members and microtubules.** (A) Primary structure of chTOG, TACC3, clathrin and GTSE1, showing the interactions between each protein (dashed lines). ACID, Aurora-A and clathrin interaction domain; CHC, clathrin heavy chain; CLC, clathrin light chain; TD, trimerization domain. The TOG domains of chTOG are numbered. Interactions were mapped previously (Gutiérrez-Caballero et al., 2015; Hood et al., 2013; Burgess et al., 2018; Rondelet et al., 2020). (B) Proposed topology of the complex on a microtubule (yellow). TACC3 and clathrin bind each other and form a composite microtubule-interaction surface. GTSE1 and chTOG bind to clathrin and TACC3, respectively. Both proteins can interact with microtubules: chTOG in a domain between TOG4 and TOG5 (Widlund et al., 2011), GTSE1 in a diffuse region between residues 161–638, although neither interaction is necessary for the complex to bind microtubules.

Inducible relocalization is a powerful method to investigate protein–protein interactions in cells and to pinpoint where and when they occur. We generated a number of cell lines to study the interactions between members of the TACC3–chTOG–clathrin–GTSE1 complex on mitotic spindles at metaphase. This approach showed that TACC3 and clathrin are core complex members, while chTOG and GTSE1 are ancillary. Our current picture of this multiprotein complex is outlined in Fig. 8.

It has been reported that PIK3C2A is a component of the TACC3–chTOG–clathrin–GTSE1 complex, where it has been proposed to act as a scaffold protein binding both TACC3 and clathrin (Gulluni et al., 2017). This proposal is consistent with several observations. First, PIK3C2A has been found to interact with clathrin, GTSE1 and TACC3 in a proteomic analysis of immunoprecipitations with each of these three proteins from mitotic lysate (Hubner et al., 2010). In that study, immunoprecipitation of PIK3C2A brought down clathrin, GTSE1 and components of the membrane trafficking machinery, but notably neither TACC3 nor chTOG co-immunoprecipitated with PIK3C2A. Second, PIK3C2A binds clathrin heavy chain via an N-terminal region that contains a clathrin box-like motif (LLLDD; Gaidarov et al., 2001). These motifs bind to grooves in the seven-bladed β-propeller that constitutes the N-terminal domain of clathrin heavy chain (Smith et al., 2017). The N-terminal domain is required for clathrin–TACC3 to localize at the spindle (Royle et al., 2005), and mutations in one of the grooves is sufficient to reduce spindle binding (Hood et al., 2013). However, while the proposal that PIK3C2A is a component of the complex makes sense, we found no evidence to suggest that PIK3C2A was even present on mitotic spindles. GFP-tagged PIK3C2A was found in clathrin-coated vesicles, as expected, but was absent from the mitotic spindles of HeLa cells. We also found that the PIK3C2A antibody used in the original study to detect the protein at the spindle gave a false signal that remained after knockout and/or knockdown of PIK3C2A. Finally, a PIK3C2A-null cell line we generated had no mitotic delays, and all members of the TACC3–chTOG–clathrin–GTSE1 complex had normal localization. We conclude that PIK3C2A is not a component of the complex and any mitotic function for this protein is doubtful. PIK3C2A has a well-established role in clathrin-mediated membrane traffic (Posor et al., 2013), and it seems likely that the presence of PIK3C2A among other membrane-trafficking factors in the original proteomic work was due to association with a fraction of clathrin that was not associated with the spindle, or erroneous binding during purification (Hubner et al., 2010).

Recent work has shown that GTSE1 contains five conserved LIDL motifs – an intrinsically disordered C-terminal region that can bind to the N-terminal domain of clathrin heavy chain (Rondelet et al., 2020). In agreement with this, we found that these motifs are redundant and that mutations reducing the total number of motifs to below three significantly impaired spindle binding. We also found that GTSE1 was an ancillary protein not required for the localization of the complex on microtubules and that inducing its mislocalization did not affect the other complex members. This interpretation is consistent with other work on GTSE1 (Rondelet et al., 2020; Bendre et al., 2016). It is a mystery why mutation of one groove of the N-terminal domain of clathrin heavy chain results in loss of the complex from the spindle, since it appears that this domain recruits GTSE1 to k-fibers but that GTSE1 is not needed for localization of the complex (Hood et al., 2013). One explanation is that this groove interacts directly with microtubules and that GTSE1 may also bind other sites on the N-terminal domain of clathrin heavy chain. Another is that the GTSE1–clathrin interaction may be important for the formation of the complex, but not for its stability once loaded onto microtubules.

The ancillary nature of GTSE1 and chTOG binding to the complex via association with clathrin and TACC3, respectively, is intriguing. Especially because GTSE1 and chTOG each have the ability to bind microtubules themselves (Monte et al., 2000; Spittle et al., 2000). Rondelet et al. have proposed that clathrin–TACC3 could be forming a ‘scaffold’ for the recruitment of other factors, such as GTSE1, to the spindle so that they can in turn perform specific functions (Rondelet et al., 2020; Bendre et al., 2016). Our work is consistent with this idea, that clathrin–TACC3 are core to spindle microtubule binding and that other ancillary factors may be recruited through this complex. In this work, we mapped a constitutive microtubule-binding region in GTSE1 to residues 161–638, whereas chTOG likely binds the microtubule lattice through a region between TOG4 and TOG5 domains. The criterion for binding clathrin–TACC3 at the spindle may include the ability to bind microtubules, which would explain the selectivity for ancillary partners and mean that clathrin adaptors, for example, are not recruited to the spindle.

Our work establishes that, in order to disrupt the TACC3–chTOG–clathrin–GTSE1 complex, agents that target (1) the TACC3–clathrin interaction or (2) the interface between TACC3–clathrin and microtubules are required. In the first case, preventing the helix that is formed by phosphorylation of TACC3 on serine 558 from binding to the helical repeat in the ankle region of clathrin heavy chain is predicted to disrupt the complex (Burgess et al., 2018). To address the second case, the microtubule interface needs to be mapped at high resolution using cryo-electron microscopy. The endogenously tagged cell lines we have developed will be useful for investigating these interactions. Besides fluorescence microscopy and knocksideways, the cells are well suited for visualizing proteins at the ultrastructural level using inducible methodologies such as FerriTagging (Clarke and Royle, 2018).

## MATERIALS AND METHODS

### Molecular biology

The following plasmids were available from previous work: MitoTrap (pMito-mCherry-FRB), dark MitoTrap (pMito-mCherryK70N-FRB), rapalog-sensitive MitoTrap (pMito-mCherry-FRB-T2098L), mCherry–α-tubulin, mCherry–CLTA (mCherry–LCa), mCherry–TACC3, chTOG–GFP, and pBrain-chTOG-GFP-shchTOG (Booth et al., 2011; Cheeseman et al., 2013; Hood et al., 2013; Wood et al., 2017; Clarke and Royle, 2018). Plasmid to express chTOG–mCherry was made by ligating a BamHI–NotI fragment from chTOG–GFP into pmCherry-N1 (made by substituting mCherry for EGFP in pEGFP-N1 at NheI and NotI). For tdTomato–GTSE1, a GFP–GTSE1 construct was first made by PCR of human GTSE1 (IMAGE: 4138532) with the addition of EcoRI–BamHI and cloning into pEGFP-C1 (Clontech) and then ligating the EcoRI–BamHI fragment from GFP–GTSE1 into ptdTomato-C1 (made by substituting tdTomato for EGFP in pEGFP-C1 at NheI and XhoI). Note that our GTSE1 constructs use the 720 residue isoform as their basis. Therefore our residue numbers differ from other work that uses the 739 residue isoform with an alternative start codon as the full-length GTSE1 (Rondelet et al., 2020). For GFP–PIK3C2A a ScaI–BstEII fragment from full-length human PIK3C2A in PCR-XL-Topo (IMAGE: 8322710) was cloned into pEGFP-C1. The mCherry-tagged GTSE1 constructs were made by PCR amplification of GTSE1 (IMAGE: 4138532) followed by insertion into pmCherry-N1 between SalI and BamHI, and using site-directed mutagenesis to introduce each mutation.

### Cell culture

HeLa cells (Health Protection Agency/European Collection of Authenticated Cell Cultures, #93021013) were maintained in Dulbecco's Modified Eagle's Medium (DMEM) supplemented with 10% FBS and 100 U ml*^−^*^1^ penicillin/streptomycin in a humidified incubator at 37°C and 5% CO_2_. Cell cultures were checked for mycoplasma contamination at six-week intervals.

Knock-in cell lines were generated by CRISPR/Cas9 gene editing. The orientation of tags (N-terminal or C-terminal) was guided by previous work on CLTA (Doyon et al., 2011), TACC3 (Cheeseman et al., 2013), chTOG (Gutiérrez-Caballero et al., 2015) and GTSE1 (Scolz et al., 2012). Briefly, HeLa cells were transfected with a Cas9n D10A nickase plasmid [pSpCas9n(BB)-2A-Puro, pX462; Addgene #48141] and a repair template. The following guide pairs were used: CLTA–FKBP–GFP cell line (guide 1, 5′-CACCGCAGATGTAGTGTTTCCACA-3′; guide 2, 5′-CACCGTGAAGCTCTTCACAGTCAT-3′), GFP–FKBP–TACC3 cell line (guide 1, 5′-CACCGGCACGACCACTTCCCACAC-3′; guide 2, 5′-CACCGACGTCTGTGTCTGGACAATG-3′), chTOG–FKBP–GFP cell line (guide 1, 5′-CACCGAAGATCCTCCGACAGCGATG-3′; guide 2, 5′-CACCGCCAGACCACATCGCTGTCGG-3′), FKBP–GFP–GTSE1 cell line (guide 1, 5′-CACCGGGAGCTCAGGTCTATGAGC-3′; guide 2, 5′-CACCGTGAGGCTGACAAGGAGAACG-3′). Details of the repair templates are available (see Data availability). Ten days after transfection, single GFP-positive cells were selected by fluorescence-activated cell sorting (FACS), expanded and validated using microscopy, western blotting, PCR and DNA sequencing. The PIK3C2A-knockout cell line was generated by transfecting HeLa cells with pSpCas9(BB)-2A-GFP (pX458; Addgene #48138) into which a single guide (5′-CACCGAGCACAGGTTTATAACAAGC-3′) had been cloned. GFP-positive cells were isolated by FACS and then single cell clones were validated using western blotting and genome sequencing. Briefly, a genomic region encompassing the target site was amplified (forward primer, 5′-CCAGTTGTGTCAGGAAATGGG-3′; reverse primer, 5′-TCCAAATCAGTCCTTGCTTTCCC-3′) and TA-cloned into pGEM-T Easy vector (Promega). Ten bacterial transformants were picked and sequenced, revealing a 1:1 ratio of the two alleles, shown in Fig. S7.

For knockdown of endogenous GTSE1 in spindle recruitment experiments, CLTA–FKBP–GFP CRISPR knock-in HeLa cells were transfected with 100 nM siRNA targeting the 3′UTR of GTSE1 [GTSE1, 5′-GCCTGGGAAATATAGTGAAACTCCT-3′; GL2 (control), 5′-CGTACGCGGAATACTTCGA-3′]. For knockdown of PIK3C2A in HeLa, RNAi was performed by transfecting 60 nM siRNA [siPIK3C2A ‘1’, 5′-GAAACTATTGCTGGATGACAGT-3′; GL2 (control), 5′-CGTACGCGGAATACTTCGA-3′], using Lipofectamine 2000 (Invitrogen), according to the manufacturer's instructions.

For DNA plasmid transfections, cells were transfected with a total of 1000–1500 ng DNA in 35 mm fluorodishes or 6-well plates using Genejuice, as per the manufacturer's instructions (Merck Millipore). Cells were typically imaged 48 h after transfection. For knocksideways experiments, cells were transfected with plasmids to express MitoTrap alone or dark MitoTrap in combination with other constructs as indicated. For the expression of GTSE1–mCherry mutants, cells were transfected with DNA 24 h after siRNA treatment using Genejuice (Merck Millipore), following the manufacturer's protocol. Cells were fixed 64 h after siRNA transfection and 40 h after DNA transfection.

Knocksideways was via the application of rapamycin (Alfa Aesar) to a final concentration of 200 nM; either live on the microscope or, in the case of immunofluorescence experiments, for 30 min prior to fixation. Successful relocalization in edited cells depends on the optimal expression of MitoTrap and the efficient application of rapamycin to cells. For Aurora-A inhibition, MLN8237 (Apexbio) was added at a final concentration of 300 nM for 40 min.

### Immunofluorescence

For immunofluorescence, cells were fixed at room temperature using PTEMF (20 mM PIPES, pH 6.8, 10 mM EGTA, 1 mM MgCl_2_, 0*.*2% Triton X-100 and 4% paraformaldehyde) for 10 min and permeabilized at room temperature in 0*.*5% Triton-X100 in phosphate-buffered saline (PBS) for 10 min. Cells were blocked in 3% BSA in PBS for 30 min. Cells were incubated for 1 h at room temperature with primary antibodies as follows: rabbit anti-α-tubulin (PA5-19489, Invitrogen; 1:1000), mouse anti-α-tubulin (B-5-1-2; Sigma; 1:1000), mouse anti-CHC (X22; CRL-2228, ATCC; 1:1000), rabbit anti-CKAP5 (PA5-59150, Thermo Fisher Scientific; 1:400), rabbit anti-chTOG (Fig. 7 only; 34032, QED Biosciences; 1:5000), mouse anti-TACC3 (ab56595, Abcam; 1:1000), mouse anti-GTSE1 (H00051512-B01P, Abnova; 1:1000) and rabbit anti-PIK3C2A (22028-1-AP, Proteintech; 1:1000). Cells were washed three times with PBS, then incubated for 1 h with Alexa Fluor 568- or Alexa Fluor 647-conjugated secondary antibodies (Molecular Probes). Finally, coverslips were rinsed with PBS and mounted with Mowiol containing DAPI (Sigma). In some experiments it was necessary to boost the GFP signal of the knock-in cells. To do this, GFP-booster (Alexa Fluor 488, Chromotek) or GFP rabbit anti-Tag, Alexa Fluor 488 (Invitrogen) 1:200 was used during the primary incubations.

### Biochemistry

For western blotting, cell lysates were prepared by scraping cells in RIPA buffer containing cOmplete EDTA-free protease inhibitor cocktail (Sigma-Aldrich), incubation on ice for 30 min and clarification in a benchtop centrifuge (20,800 ***g***) for 15 min at 4°C. Lysates were boiled in 4× Laemmli buffer for 10 min and resolved on a precast 4–15% polyacrylamide gel (Bio-Rad). Proteins were transferred to nitrocellulose using a Trans-Blot Turbo Transfer System (Bio-Rad). Primary antibodies used were mouse anti-α-tubulin (DM1A, Sigma) 1:10,000, rabbit anti-CLTA (sc-28276, Santa Cruz) 1:1000, goat anti-TACC3 (AF5720, Novus Biologicals) 1 µg ml*^−^*^1^, rabbit anti-chTOG (34032, QED Biosciences) 1:2000, mouse anti-GTSE1 (H00051512-B01P, Abnova) 1:500 and rabbit anti-PIK3C2A (22028-1-AP, Proteintech) 1:1000. Secondary antibodies used were anti-mouse, -rabbit and -goat IgG HRP conjugates. For detection, enhanced chemiluminescence detection reagent (GE Healthcare) and manual exposure of Hyperfilm (GE Healthcare) was performed.

### Microscopy

For live-cell imaging, medium was changed to Leibovitz (Gibco) L-15 CO_2_-independent medium supplemented with 10% FBS. Imaging was performed on a Nikon Ti epifluorescence microscope with standard filter sets and 100× or 60× (both 1.4 NA, oil, PlanApoVC) objectives, equipped with a heated environmental chamber (OKOlab) and either a CoolSnap MYO or 95B Prime camera (Photometrics), using NIS elements AR software.

For overnight mitotic imaging, asynchronously growing cells were incubated with 0*.*5 µM SiR-DNA (Spirochrome) for 60 min to visualize DNA. Image stacks (7×2 µm optical sections; 1×1 binning) were acquired every 3 min for 12 h with a 40× oil-immersion 1.3 NA objective using an Olympus DeltaVision Elite microscope (Applied Precision, LLC) equipped with a CoolSNAP HQ2 camera (Roper Scientific). Images were acquired at 10% neutral density using a Cy5 filter and an exposure time of 100 ms. A stage-top incubator maintained cells at 37°C and 5% CO_2_, with further stabilization from a microscope enclosure (Weather station, PrecisionControl) held at 37°C. To analyze mitotic progression after knocksideways, cells were transfected with mCherry–MitoTrap(T2098L), and asynchronously growing cells were treated (or not) with 1 µM rapalog AP21967 prior to 30 min SiR-DNA labeling and overnight imaging. Rapalog was used for these experiments because we found that rapamycin treatment affected mitotic timing, whereas in parental HeLa cells, control versus rapalog-treated timings were unaffected (nuclear envelope breakdown-to-metaphase: 13.2 min versus 12.6 min, respectively; metaphase-to-anaphase: 20.8 min versus 20.6 min, respectively).

To image fixed cells, image stacks (5×1 µm optical sections) were acquired on a spinning-disk confocal system. Either an Ultraview (Perkin Elmer) system or a Nikon CSU-W1 spinning-disk confocal with SoRa upgrade was used with a 60×1.4 NA oil-immersion objective (Nikon) and Hamamatsu Orca-R2 (Ultraview) or 95B Prime (Photometrics) camera.

### Data analysis

Analysis of knocksideways movies was done by extracting a pre- and a post-rapamycin multichannel image from the sequence. An automated procedure in Fiji (https://imagej.net/Fiji) measured three regions in each of the following areas: spindle, cytoplasm and mitochondria, after registration of the pre- and post-rapamycin images. A background measurement and a whole cell fluorescence measurement were also taken. The average value for each region, after background subtraction, was corrected for bleach using the whole cell fluorescence measurement (background-subtracted) for the respective channel. Data were exported in csv format and read into IgorPro (WaveMetrics), where a custom-written procedure analyzed the data and generated all the plots. Ternary diagrams of spindle, mitochondria and cytoplasm fluorescence revealed that knocksideways resulted in movement mainly between spindle and mitochondria (Fig. S8). Therefore, the fraction of fluorescence at the spindle and mitochondria were used to generate the arrow plots.

For spindle localization analysis of fixed cells, a 31×31 pixel (1.4 µm^2^) region of interest was used to measure three regions of the spindle, the cytoplasm and one region outside of the cell as background, using Fiji. Following background subtraction, the average spindle fluorescence was divided by the cytoplasm fluorescence to give a measure of spindle enrichment. To quantify the microtubule localization of GTSE1 fragments, a line-scan analysis method adapted from Hooikaas et al. (2019) was used. Using an automated procedure in Fiji, average fluorescence intensities from three lines, 1–3 µm length, along microtubules stained for α-tubulin and three adjacent lines (not coincident with microtubules) were measured. Following background subtraction, the average fluorescence intensity of the microtubule line scan was divided by the average fluorescence intensity of the adjacent control line scan to generate a microtubule enrichment ratio. Analysis was done by an experimenter blind to the conditions of the experiment. All figures were made in Fiji, R or Igor Pro 8 and assembled using Adobe Illustrator. All code used in this article is available at https://github.com/quantixed/p053p030.

## Supplementary Material

Supplementary information

Reviewer comments
