# Supplementary material for: Defining endogenous TACC3–chTOG–clathrin–GTSE1 interactions at the mitotic spindle using induced relocalization
Source: J Cell Sci. 2021 Feb 1;134(3):jcs255794. doi: 10.1242/jcs.255794 (PMC7875487; doi:10.1242/jcs.255794)
Supplement: Supplementary information [file joces-134-255794-s1.pdf]

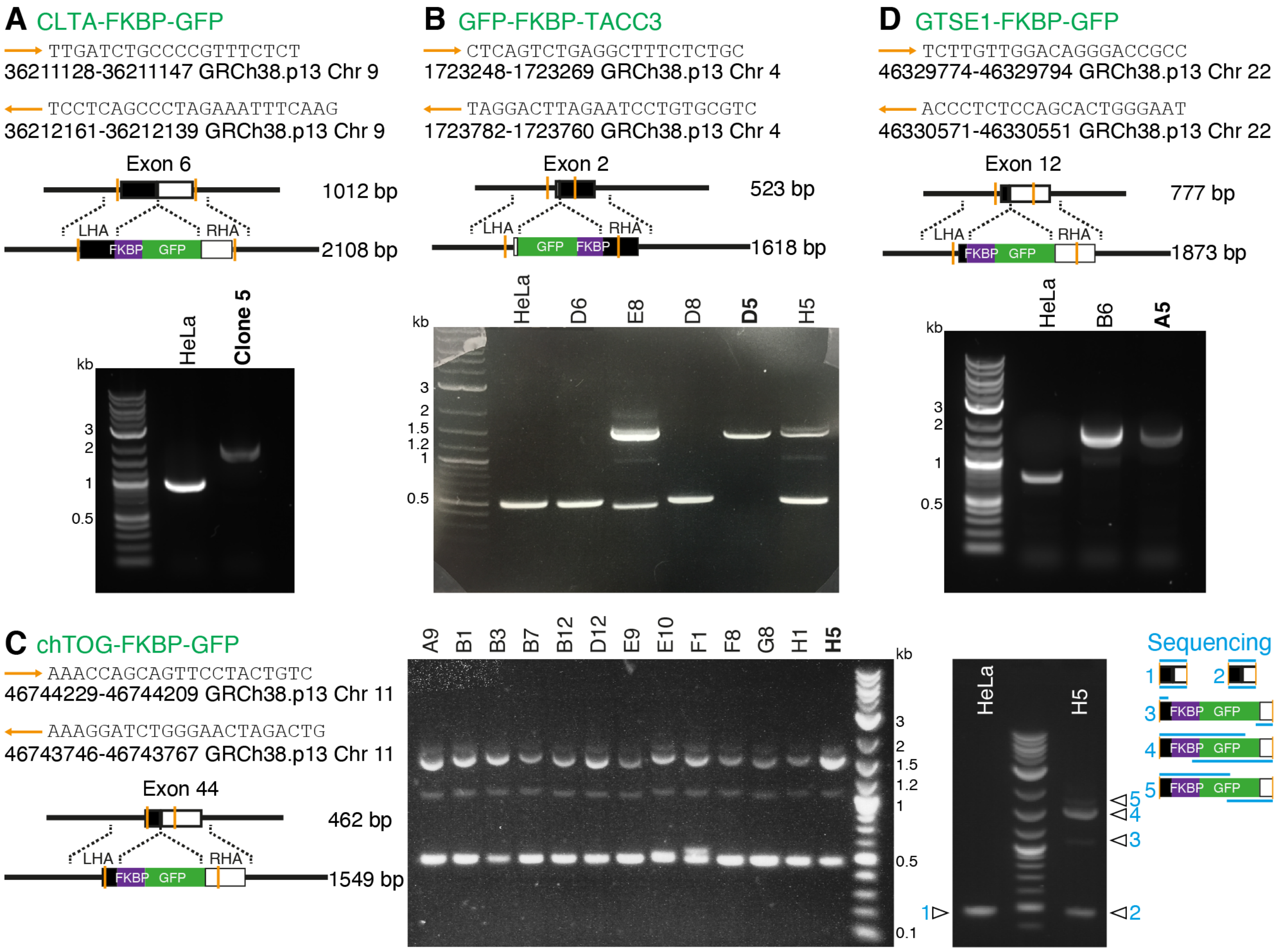

Figure S1: Genotyping gene-edited cell lines

PCR analysis to confirm insertion of tandem FKBP-GFP tag at each locus. (**A-D**) For each indicated cell line, a schematic diagram of the gene targeting event is shown with primers that anneal to left and right homology arms (orange lines); the amplicon for wild-type and successful insertion of tandem tag is shown. SyBr-stained agarose gels of PCR from genomic DNA isolated from parental HeLa or from recovered clones. Bold labels indicate the clone used in this study. (**C**) A sample of recovered clones displaying heterozygosity. Sequencing of amplicons from clone H5 revealed tagged and untagged alleles.

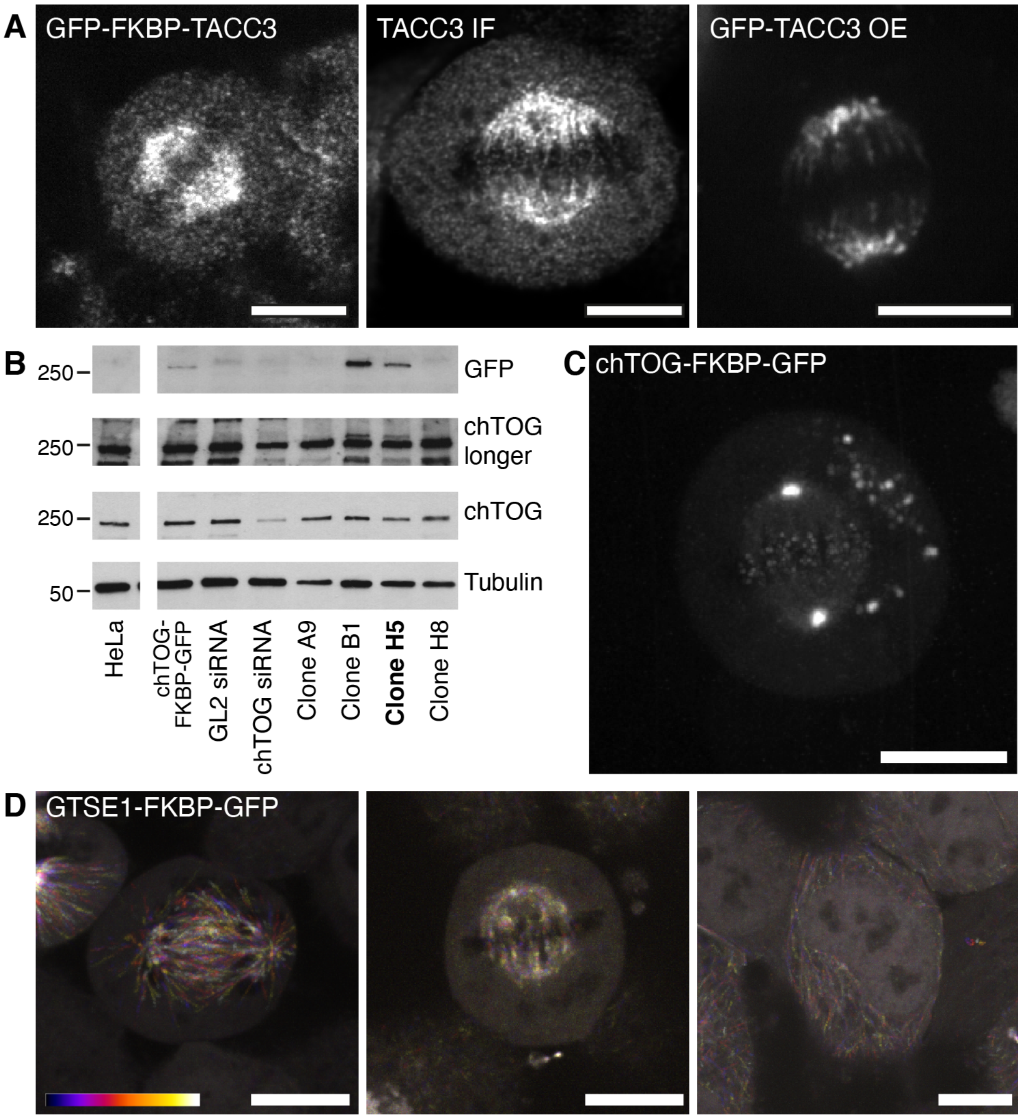

Figure S2: Further validation of gene-edited cell lines

(**A**) Representative confocal micrographs of the GFP-FKBP-TACC3 cell line (with GFP booster), anti-TACC3 immunofluorescence in unedited HeLa cells, and over-expression of GFP-TACC3 in unedited HeLa. (**B**) Western blots of four representative chTOG-FKBP-GFP clones. For comparison, parental HeLa are shown, untransfected (HeLa) or expressing chTOG-FKBP-GFP, or transfected with siRNA as described. A single gel is shown probed with a chTOG antibody (two different exposures), tubulin as a loading control, the upper blot was reprobed with a GFP antibody. The chTOG antibody does not appear to detect the tagged protein with the same efficiency as the unedited protein. (**C**) Maximum intensity projection of a stack of confocal images of a live chTOG-FKBP-GFP knock-in cell at metaphase. (**D**) Temporal color coded stacks of GTSE1-FKBP-GFP cells to show fluorescence at microtubule plus-ends. Three examples are shown of cells in anaphase (61 s), metaphase (10 s), and interphase (61 s). Scale bars, 10 µm.

Journal of Cell Science • Supplementary information

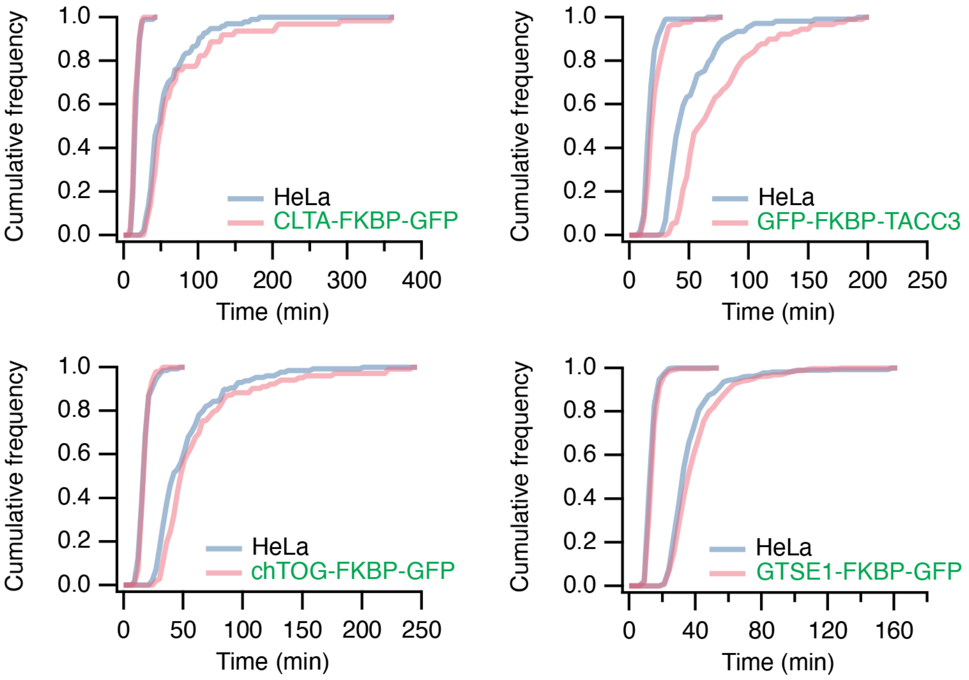

Figure S3: Mitotic progression of gene-edited cell lines

Cumulative histograms of timings from nuclear envelope breakdown (NEB) to metaphase (short duration) and NEB to anaphase (long duration). Gene-edited cells are as indicated and were imaged alongside their respective unedited parental HeLa counterpart. All imaging experiments were done three times. Number of cells analyzed (edited line and parental) = CLTA-FKBP-GFP: 62 and 97; GFP-FKBP-TACC3: 90 and 106; chTOG-FKBP-GFP: 102 and 128; GTSE1-FKBP-GFP: 265 and 319.

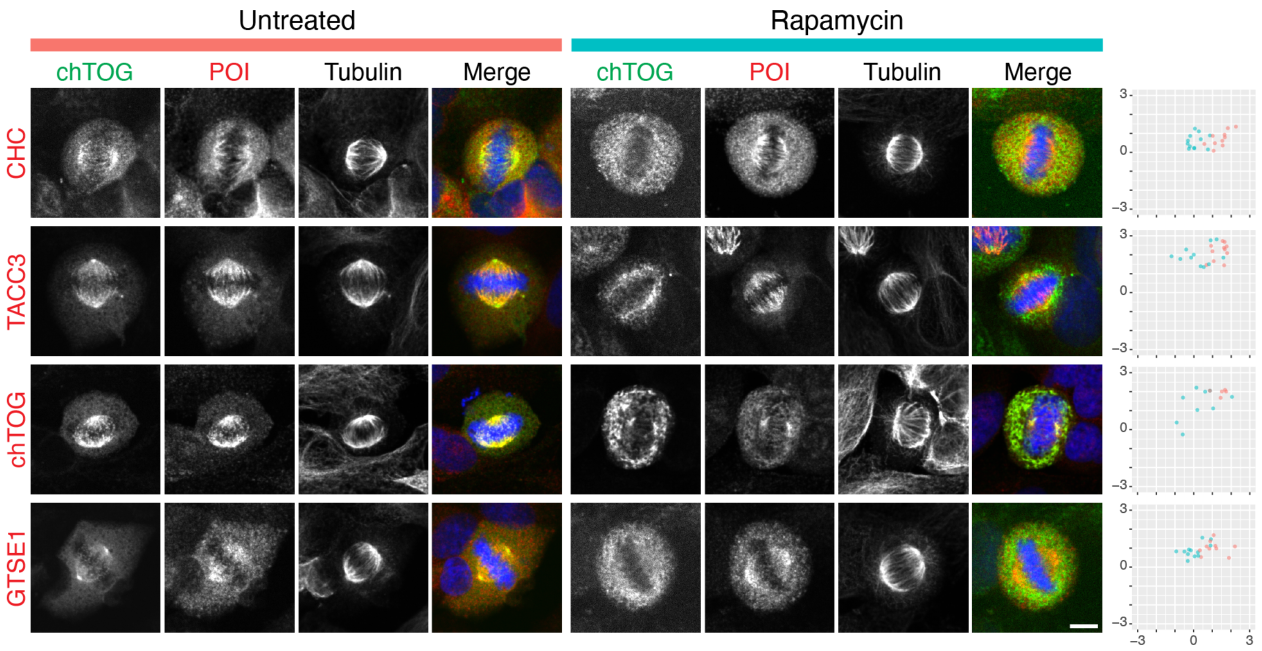

Figure S4: Verification of chTOG knocksideways results

Knocksideways experiments using transiently expressing chTOG-FKBP-GFP and dark MitoTrap in HeLa cells depleted of endogenous chTOG by RNAi. Representative widefield micrographs of cells that were treated with rapamycin (200 nM) for 30 min, fixed and stained for tubulin and either CHC, TACC3, chTOG, or GTSE1 (protein-of-interest, POI, red). Scale bar, 10 µm. Right, quantification of images. Spindle localization of the target protein (x-axis) and the protein-of-interest (y-axis) in control (salmon) and knocksideways (turquoise) cells. Spindle localization is the ratio of spindle to cytoplasmic fluorescence shown on a log2 scale (1 is twice the amount of protein on the spindle as the cytoplasm, -1 indicates half the amount on spindle versus cytoplasm). Quantification of cells from a single experiment are shown.

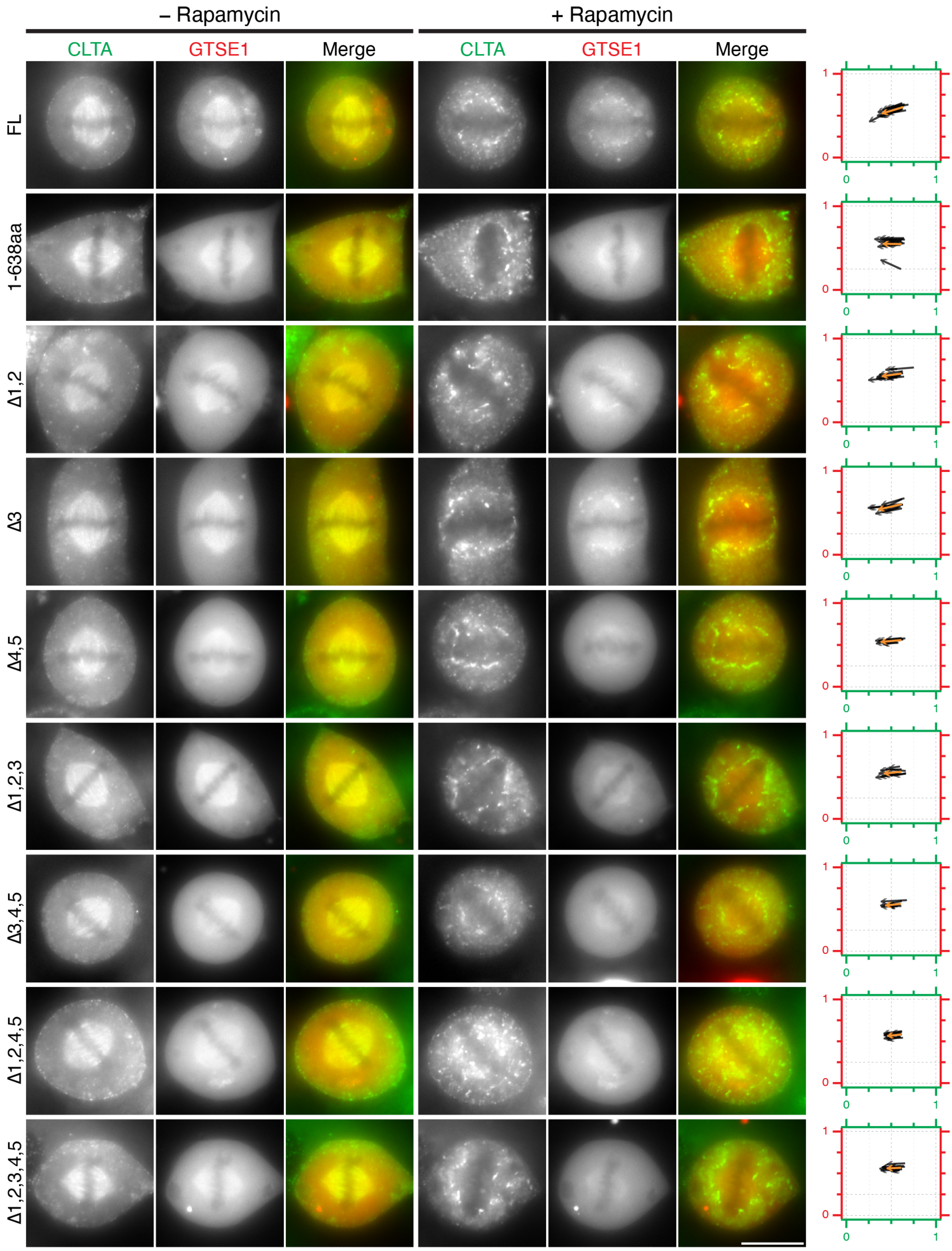

Figure S5: Live cell imaging of knocksideways in CLTA-FKBP-GFP cells expressing GTSE1 LIDL mutants. Stills from live cell imaging of clathrin knocksideways in metaphase CLTA-FKBP-GFP cells expressing the indicated GTSE1-mCherry constructs. Rapamycin (200 nM) was added to induce removal of clathrin and imaged for a total of 10 min to visualize co-rerouting of GTSE1 mutants. Scale bar, 10 µm. (Right) Quantification of GTSE1 co-rerouting shown as arrow plots. Arrows show the fraction of spindle and mitochondria fluorescence that is at the spindle (i.e. 1 = completely spindle-localized, 0 = mitochondria-localized), for both channels, moving from pre to post rapamycin localization. Black arrows represent individual cells, the orange arrow is the mean. n = 7-12 cells per condition.

Journal of Cell Science • Supplementary information

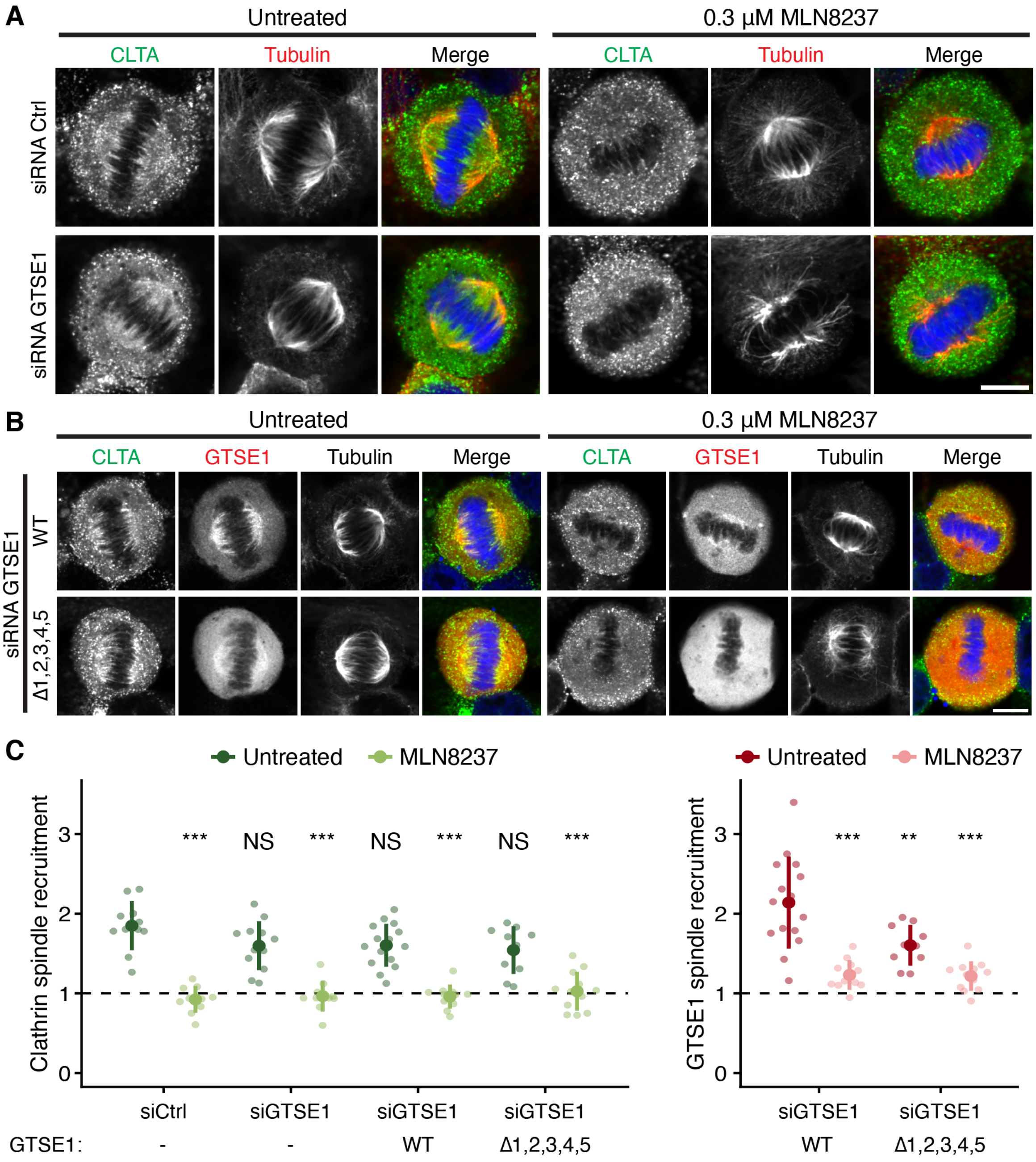

Figure S6: Comparison of GTSE1 LIDL motif ablation with the effect of Aurora-A inhibition on spindle localization of clathrin and GTSE1.

Representative widefield micrographs of CLTA-FKBP-GFP cells at metaphase to show the spindle localization of clathrin (**A**), or GTSE1-mCherry construct (WT or Δ1,2,3,4,5, red) and clathrin (**B**). Cells were treated with control (GL2, Ctrl) or GTSE1 siRNA and Aurora-A kinase was inhibited with MLN8237 (0.3 µM, 40 min) as indicated. Cells were stained for tubulin (red in A, not shown in merge in B) and DNA (blue). A GFP-boost antibody was used to enhance the signal of CLTA-FKBP-GFP (green). Scale bar, 10 µm. (**C**) Quantification of clathrin and GTSE1 spindle recruitment. Each dot represents a single cell, n = 10-15 cells per condition. The large dot and error bars show the mean and the mean ±SD, respectively. Analysis of variance (ANOVA) with Tukey's post-hoc test was used to compare the means between each group, using the untreated cells + siRNA Ctrl (clathrin) and untreated cells + WT GTSE1 (GTSE1) for comparison. The p-value level is shown compared to WT: ***, $p < 0.001$; **, $p < 0.01$; NS, $p > 0.05$.

Journal of Cell Science • Supplementary information

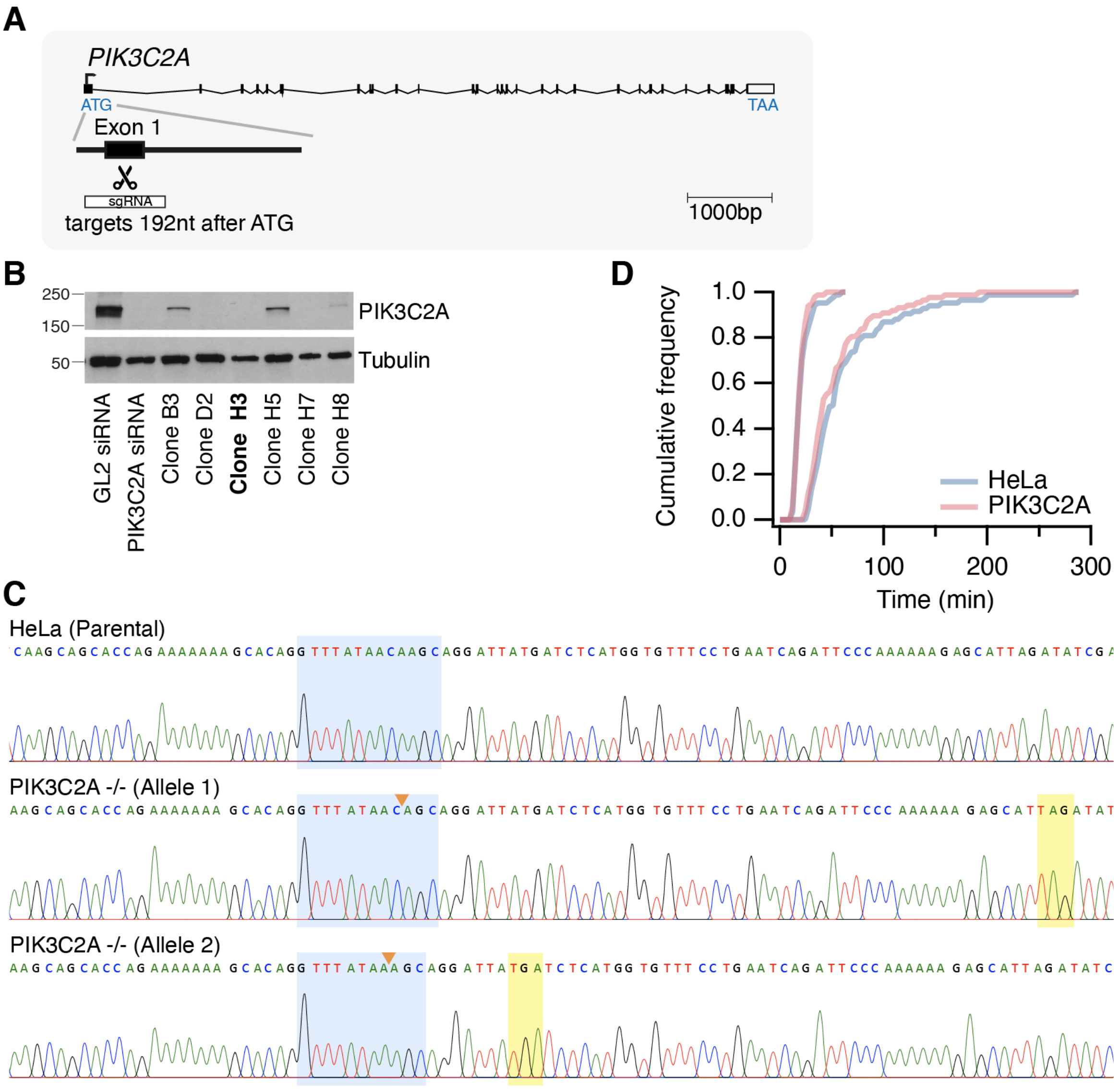

Figure S7: Generation of PIK3C2A-null HeLa cells.
(**A**) Targeting strategy for generation of a PIK3C2A-null cell line. HeLa cells were transfected with plasmid to express GFP coupled Cas9 nuclease and sgRNA targeting 192 bp from the start codon of the PIK3C2A gene. Scale bar, 1000 bp. (**B**) Western blot of a selection of clones grown after expansion of GFP-expressing cells sorted by FACS. Presence of a band for PIK3C2A was assessed compared parental HeLa cells treated with PIK3C2A siRNA or control, GL2. Tubulin, loading control. Clone H3, was used in this study (bold). (**C**) A genomic fragment from clone H3 was cloned into a cloning vector and 20 bacterial clones were picked and sequenced to assess the status of PIK3C2A alleles. We found two sequences, indicating two alleles and both had deletions (orange arrows) which resulted in premature truncation of the PI3KC2A gene after 87 and 72 residues, respectively. Stop codon is highlighted in yellow. Blue window highlights the edited region. (**D**) Mitotic progression of PIK3C2A-null cells compared to parental HeLa cells. Cumulative histograms of timings from nuclear envelope breakdown-to-metaphase (NEB-M, long duration) and metaphase-to-anaphase (M-A, short duration). Progression experiments were done three times. Number of cells analyzed = PIK3C2A$^{-/-}$: 87; parental: 84.

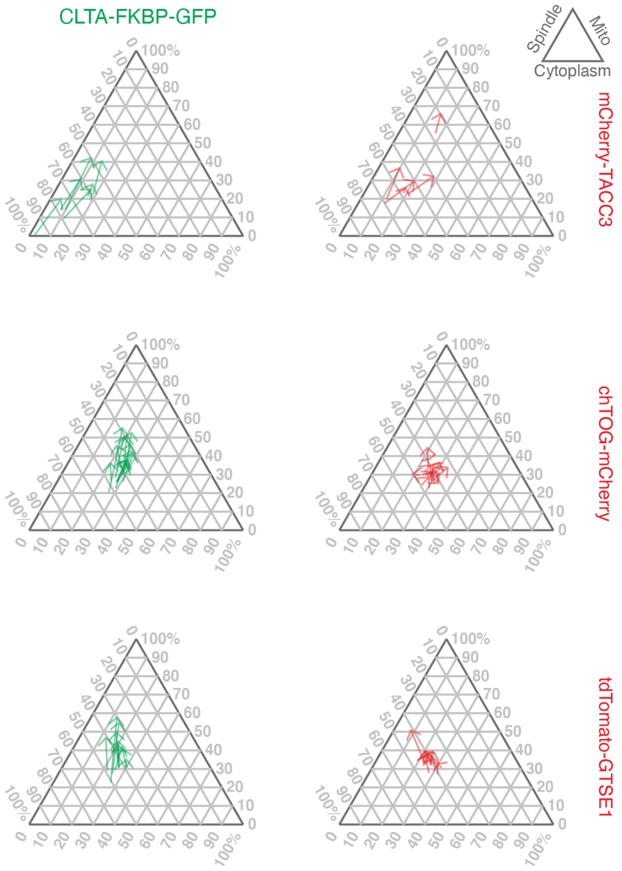

Figure S8: Ternary diagrams of live CLTA-FKBP-GFP spatial relocalization experiments.
Localization before and after addition of rapamycin is shown by an arrow for each cell. Ternary diagrams can be read using the key. For example, a protein that is localized entirely on the miochondria and is absent from the spindle would be at the top corner of the triangle. Generally, movement (if it occurs) is from the bottom left corner to the upper corner with cytoplasmic signal staying approximately constant.

Journal of Cell Science • Supplementary information

**Cell line**	Clone	Genomic status	Western band-shift	Western endogenous	Insertion	Unedited allele(s)	Expected localization	KSW	Mitosis	Notes
CLTA-FKBP-GFP	5	Homozygous	Yes	No	Yes	No	Yes	Yes	Normal	Clathrin triskelia are CLTC with CLTA and CLTB. In HeLa, CLTA > CLTB expression
GFP-FKBP-TACC3	D5	Homozygous	Yes	No	Yes	No	Yes	Yes	Slight delay	–
chTOG-FKBP-GFP	H5	Heterozygous	Yes	Yes	Yes	Yes	Yes	Yes	Normal	>20 heterozygous clones, 0 homozygous recovered from three attempts
GTSE1-FKBP-GFP	A5	Homozygous	Yes	No	Yes	No	Yes	Yes	Normal	–

Table S1: Summary of knock-in cell lines used in this study.
Details of each cell line used. Insertion and unedited allele(s) were detected using PCR and genomic sequencing. KSW, Knocksideways.

Cell line	Protein of interest			
	**clathrin**	**TACC3**	**chTOG**	**GTSE1**
CLTA-FKBP-GFP	-1.04 [-1.51, -0.673] *<br>–	-1.21 [-1.69, -0.704] *<br>-0.181 [-0.336, -0.0768] *	-1.16 [-1.55, -0.755] *<br>-0.0535 [-0.0911, -0.009] *	-0.399 [-0.733, -0.0903] *<br>-0.0991 [-0.161, -0.0547] *
GFP-FKBP-TACC3	-0.136 [-0.708, 0.375]<br>-0.0352 [-0.0568, -0.0144] *	-1.06 [-1.94, -0.267] *<br>–	-1.19 [-1.47, -0.94] *<br>-0.115 [-0.223, -0.0582] *	-0.248 [-0.62, 0.128]<br>-0.0418 [-0.0759, -0.013] *
chTOG-FKBP-GFP	-0.65 [-1.35, 0.0585]<br>-0.0331 [-0.105, 0.022]	-0.745 [-1.48, 0.314]<br>-0.0198 [-0.146, 0.0762]	-0.45 [-0.989, 0.098]<br>–	0.698 [-0.138, 2.36]<br>-0.033 [-0.0663, 0.00429]
GTSE1-FKBP-GFP	-0.267 [-0.416, -0.125] *<br>-0.00823 [-0.0356, 0.0205]	-1.17 [-1.52, -0.829] *<br>0.000224 [-0.0544, 0.0563]	-0.484 [-0.757, -0.205] *<br>-0.00675 [-0.0249, 0.0106]	-0.775 [-0.938, -0.613] *<br>–

Table S2: Summary of knocksideways experiments.
Each row is a cell line and the effect of relocalization of the tagged protein to mitochondria on the spindle localization of each protein-of-interest is indicated. Effect sizes for immunofluorescence (upper) and live cell (lower) knocksideways experiments, are presented with bias-corrected and accelerated bootstrap 95% confidence intervals. *, interval is less than 0.

Journal of Cell Science • Supplementary information

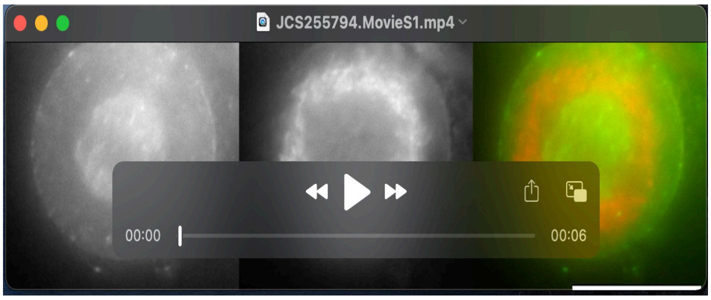

Movie 1: Knocksideways of CLTA-FKBP-GFP. Typical widefield movie of relocalization in response to rapamycin (200 nM) in cells co-expressing mCherry-MitoTrap.
Time, mm:ss. Scale bar, 10 µm.

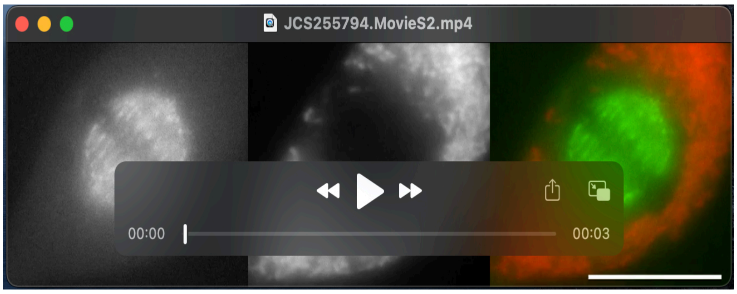

Movie 2: Knocksideways of GFP-FKBP-TACC3. Typical widefield movie of relocalization in response to rapamycin (200 nM) in cells co-expressing mCherry-MitoTrap.
Time, mm:ss. Scale bar, 10 µm.

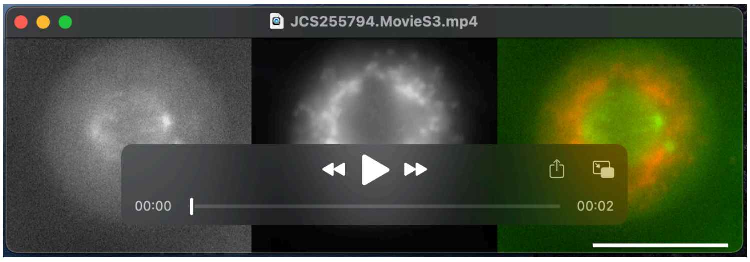

Movie 3: Knocksideways of chTOG-FKBP-GFP. Typical widefield movie of relocalization in response to rapamycin (200 nM) in cells co-expressing mCherry-MitoTrap.
Time, mm:ss. Scale bar, 10 µm.

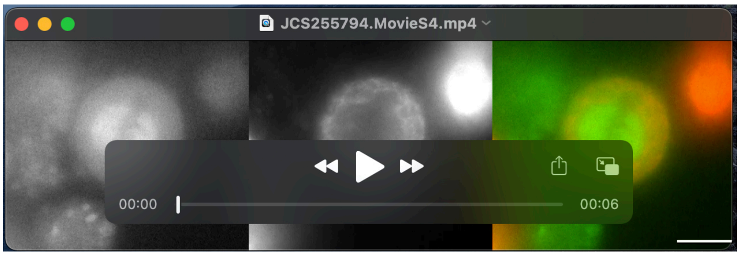

Movie 4: Knocksideways of GTSE1-FKBP-GFP. Typical widefield movie of relocalization in response to rapamycin (200 nM) in cells co-expressing mCherry-MitoTrap.
Time, mm:ss. Scale bar, 10 µm.

Journal of Cell Science • Supplementary information
