# Supplementary material for: Defining endogenous TACC3–chTOG–clathrin–GTSE1 interactions at the mitotic spindle using induced relocalization
Source: J Cell Sci. 2021 Feb 1;134(3):jcs255794. doi: 10.1242/jcs.255794 (PMC7875487; doi:10.1242/jcs.255794)
Supplement: Reviewer comments [file joces-134-255794_review_history.pdf]

Defining endogenous TACC3-chTOG-clathrin-GTSE1 interactions at the mitotic spindle using induced relocalization

Ellis L. Ryan, James Shelford, Teresa Massam-Wu, Richard Bayliss and Stephen J. Royle
DOI: 10.1242/jcs.255794

Editor: Michael Way

Review timeline

Submission to Review Commons:	3 July 2020
Original submission:	15 October 2020
Editorial decision:	16 October 2020
First revision received:	11 December 2020
Accepted:	14 December 2020

Reviewer 1

Evidence, reproducibility and clarity

Here Ryan et al. have used localization analysis following induced rapid relocalization of reproducibility endogenous proteins to investigate the composition and recruitment hierarchy of a clathrin-TACC3-based spindle complex that is important for microtubule organization and stability. The authors generate different HeLa cell lines, each with one of four complex members (TACC3, CLTA, chTOG and GTSE1) endogenously tagged with FKBP-GFP via Cas9- mediated editing. This tag allows rapid recruitment to the mitochondria upon rapamycin addition ("knocksideways"). They ultimately quantify each of the 4 components' localization to the spindle following knocksideways of each component using fluorescently-tagged transfected constructs. The authors' interpretation of the results of this analysis are summarized in the last model figure, in which a core MT-binding complex of clathrin and TACC3 recruit the ancillary components GTSE1 and chTOG. In addition, the authors investigate the contribution of individual clathrin-binding LIDL motifs in GTSE1 to the recruitment of clathrin and GTSE1 to spindles. Their findings here largely agree with and confirm a recent report regarding the contribution of these motifs to GTSE1 recruitment to the spindle. They further analyzed GTSE1 fragments for interphase and mitotic microtubule localization, and identified a second region of GTSE1 required (but not sufficient) for spindle localization. Finally, the authors report that PIK3C2A is not part of this complex, contradicting (correcting) a previously published study.

Major comments:

1. The chTOG-FKBP-GFP cell line the authors generate has only a small fraction of chTOG tagged, and thus should not be used for any conclusions about protein localization dependency on chTOG. Because they were unable to construct a HeLa cell line with all copies tagged, the authors expect that the homozygous knock-in of chTOG-FKBP-GFP is lethal, and thus their experience is appropriate to report. However, the authors should not use this cell line alone to make statements about chTOG dependency. They would have to use similar localization analysis, but after another method to disrupt chTOG (as a second- best approach), such as RNAi. In fact, they have reported this in a previous publication (Booth et al 2011). However, the result was different. There, loss of chTOG resulted in reduced clathrin on spindles, suggesting it may stabilize or help recruit the complex. Alternatively, they could remove their chTOG data, but this would compromise the "comprehensive" nature of the work.

2. The authors initially analyze complex member localization after knocksideways experiments by antibody staining, which has the advantage of analyzing endogenous proteins (versus the later

© 2021. Published by The Company of Biologists under the terms of the Creative Commons Attribution License (https://creativecommons.org/licenses/by/4.0/).

transfected fluorescent constructs). Setting aside potential artefacts from fixation, this would seem to be a better method for controlled analysis to take advantage of their setup (short of generating stable cell lines with second proteins endogenously tagged in a second color - a huge undertaking). The authors conclude that antibody specificity problems confounded their analysis and explained unusual results.
However, I think is worth investing a little more effort to sort this out, rather than bringing doubt to the whole data set. Verifying and then using another antibody for chTOG localization would be informative. Of course, the negative control should not be their chTOG- FKBP-GFP line, as it does not relocalize most of chTOG.

In the case of GTSE1, an alternative explanation to antibody specificity issues would be that the GTSE1-FKBP-GFP cell line is not in fact homozygously tagged. Given the low expression levels on the western provided, and the detection of GTSE1 on the spindle in the induced GTSE1-FKBP-GFP cell line (but not TACC3-FKBP-GFP), it seems plausible that an untagged copy remains. If there are multiple copies of GTSE1 in Hela cells, one untagged copy could represent a small fraction of total GTSE1. This should thus be ruled out. GTSE1 clones should be analyzed with more protein extracts loaded - dilutions of the extracts can determine the sensitivity of the blot to lower protein levels. In addition, sequencing of genomic DNA can reveal a small percentage with different reads.

3.There is a lot of data contained in the small graphs summarizing quantification of localization in Figs 3 and 4. They would be more accessible to the reader if they were larger and/or an "example" of the chart with labels was present explaining it (essentially what is in the figure legends). Furthermore, there is no statistical test applied to this data that I see.
This is needed. How do authors determine whether there is an "effect"?

Minor issues:

1. The GTSE1 constructs used for mutation and localization analysis are 720 amino acids long. A recent study analyzing similar mutations uses a 739 amino acid construct (Rondelet et al 2020). The latter is the predominant transcript in NCBI and Ensembl databases. It appears the construct used by the authors omits the first 19 a.a.. I do not think using the truncated transcript affects conclusions of the manuscript, but it could generate confusion when identifying residues based on a.a.#s of mutant constructs (Fig 6). This should be somehow clarified.

2. The labeling of constructs in Fig 6C/D is confusing, and appears shifted by eye at places. Please relabel this more clearly.

The recommended new experimental data (Analysis complex member levels on spindles after full perturbation of spindle chTOG; new chTOG antibody stainings in the FKBP lines; reanalysis of GTSE1 DNA/protein in GTSE1-FKBP line) should only require a new antibody/siRNA, plus a few weeks time to repeat the analyses already in the paper with new reagents.

Significance

While multiple individual components of this complex have been previously characterized, the structure and nature of the complex formation and its recruitment to microtubules/spindles remains a complex problem that has yet to be solved.

Overall this study represents a comprehensive localization-dependency analysis of the Clathrin-TACC3 based spindle complex using a consistent methodology. Although several of the conclusions of the findings echo previous reports, some of the previous literature is contradictory within itself as well as with the conclusions here. Analyzing all components with a single, rapid-perturbation technique thus has great value to present a clear data set, given that the experimental setup conditions and analysis are solid (a goal to which the majority of comments refer).

Beyond the complex localization/recruitment analysis, two novel findings of this study that emerge are:

© 2021. Published by The Company of Biologists under the terms of the Creative Commons Attribution License (https://creativecommons.org/licenses/by/4.0/).

a) GTSE1 contains a second, separate protein region, distinct from the clathrin-binding motifs that is required for its localization to the spindle, and most likely a microtubule-interaction site. This suggests that GTSE1 recruitment to the spindle is more complex than previously reported.

b) PI3KC2A, which has been reported previously to be a stabilizing member of this complex, is in fact not a member, nor localizes to spindles, nor displays a mitotic defect after loss. This is important conclusion to be made as it would correct the literature, and avoid future confusion.

Reviewer 2

Evidence, reproducibility and clarity

In this paper, the authors investigate the nature of interactions between members of the TACC3-chTOG-clathrin-GTSE1 complex on the mitotic spindle. By using a series of HeLa cell lines that they have created by CRISPR/Cas9 editing to enable spatial manipulation (knocksideways) of either TACC3, chTOG, clathrin and GTSE1, they show that on spindle microtubules TACC3 and clathrin represent core complex members whereas chTOG and GTSE1 bind to them respectively but not to each other. Additionally, the authors find that the protein PIK3C2A, which has been implicated in this complex previously is in fact not a component of this complex in mitotic cells. The main advance of the paper in my opinion is the endogenous tagging of the proteins for knocksideways experiments since former experiments depended on RNAi silencing and expression of tagged proteins from plasmids, which introduced issues of protein silencing efficiency and plasmid overexpression problems. This approach seems to alleviate these problems, except in the case of chTOG which seems to be lethal in its homozygous variant.

Major comments:

I find the key conclusions regarding the localization of the components of the complex convincing. There are some issues regarding the specificity of antibodies in immunostaining experiments (Fig 3.) and the influence of mCherry-TACC3 expression on distorted localization of the complex prior to knocksideways. However, I think the general conclusion about which complex components (clathrin and TACC3) influence the localization of the other proteins in the complex (chTOG and GTSE1) stands. One thing that I miss from the paper is the data on the consequences on the spindle shape and morphology after knocksideways. I have noticed on images in both Figure 3 and Figure 4 that in some cases distribution of the signal seems to influence quite a bit the spindle morphology. Also, In Figure 3 I have noticed what seems to me a quite big variation in spindle size in tubulin signal in both untreated and rapamycin cells. Since authors have many of these images already, I believe it would be realistic, not costly and of additional value for the paper to provide more data on the consequences of the knocksideways experiments. Change of spindle size, tubulin intensity and DNA/kinetochore misalignment upon knocksideways would be helpful to appreciate more the findings of the paper. More so since the authors on more than one occasion find their motivation in the field of cancer research and spindle stability relation to it. Some data connection to this motivation would be of value. Experiments seem reproducible.

Minor comments:

I have some problems with the clarity of Figure 3 and 4. For Figure 3. In Figure 3 plots on the right are a bit small and not easy to read. Some reorganization of the figure might be beneficial. In Figure 4 plots to the right are also too small to be clear. Also, I miss the number of cells (n) I can't see the number of individual arrows because of the size of graphs.

Significance

I find that the biggest significance of the paper is in the creation of new tools (cell lines) to study the localization of proteins TACC3, chTOG, clathrin and GTSE1. Cell lines where endogenous proteins can be delocalized rapidly will be of value for scientist working not only in mitosis but such as in the case of clathrin research, vesicle formation and trafficking or p53-dependent apoptosis in the case of GTSE1. In the field of mitosis it will surely help and speed up the research concerning the role of these proteins in spindle assembly and stability.

© 2021. Published by The Company of Biologists under the terms of the Creative Commons Attribution License (https://creativecommons.org/licenses/by/4.0/).

Field of expertise: mitotic spindle

Reviewer 3

Evidence, reproducibility and clarity

Summary:

This papers analyses the chTog/TACC3/clathrin/GTSE1 complex that crosslinks and stabilises microtubule bundles in the mitotic spindle. The authors have developed an elegant knock sideways approach to specifically analyse the effects of removing individual components of the complex from the spindle and study the effect this has on the other interactors. They report, based on these assays that the core of the complex is formed by TACC3 and Clathrin while GTSE1 and chTog are auxiliary interactors. They also refute previous evidence that this complex also incorporates PIK3C2A. Overall, this is an interesting study that distinguishes itself predominantly by its methodology. However, some of the reported results need more thorough analysis to allow convincing conclusions.

Major comments:

1) The knockside way method is the main highlight if this paper. Unlike previous studies by the PI, this time endogenous genes are tagged which is a key advance and allows much better interpretation of the results. I am not sure why the authors have chosen HeLa cells as their model here, given the messed up genome of these cells. A non-transformed cell line would have been preferable, but as a proof of principle study, I think HeLa are acceptable, and I wouldn't expect the authors to repeat all the experiment in another system.
Figure 1,2 and S1 are describing and validating this approach in some detail, but this will require some more work.
The authors state that gene targeting was validated using a combination of PCR, sequencing, Western blotting, but show only the results for westerns. PCR analysis that demonstrates homozygous or heterozygous gene targeting should be shown here.
Another issue is the penetrance of the phenotypes induced by Rapamycin. The authors show nice data of the system working in individual cells but do not give us an idea if this happens in all cells. The localisation of the individual tagged genes should be quantified (ideally with line plots) in 50 randomly chosen mitotic cells with 3 repeats before and after rapamycin treatment. Moreover, the analysis of mitotic duration (Figure S1D) should be extended to include a plus Rapamycin cohort and this should be moved in the main Figure. If the system works only in a small proportion of cells, this should be clearly stated. I don't think this would prevent publication, but it is an important piece of information that is missing.

2) Apart from a simple quantification of mitotic duration, I believe a more detailed mitotic phenotype analysis for each knock-side way gene, especially the homozygous targeted clones, should be included. This can involve more high-resolution live cell imaging of mitotic progression with SiR-DNA and GFP-tubulin, using the dark mitotrap.

3) Overall, the quantitative analysis in Figure 3 ,4 and 7 is not good enough and sometimes doesn't fully support the conclusions. In Figure 3,4 a convoluted way of demonstrating the change in localisation is shown and this panel is so small that is almost impossible to read. Also, there is no statistical analysis, and the sample size seems very small . At least 25 cells should be analysed here in 3 repeats. I would suggest to unify the quantification in the MS and use the line plots shown in Figure 5 and 6 and compare each protein before and after rapamycin addition. This is much easier to read and more convincing. The images of the cells panels can be moved to a supplement as they contain very little information. This would generate space to expand the size and depth of the quantitative analysis. Instead of Anova tests, I would recommend using a simple t-test comparing each condition to its relevant control since this is the only relevant comparison in the experiment. Statistical significance should be calculated for each experiment with sufficient sample size. It would also be better to show the individual data points from the three repeats in different colours so that the reproducibility between repeat can be judged.

© 2021. Published by The Company of Biologists under the terms of the Creative Commons Attribution License (https://creativecommons.org/licenses/by/4.0/).

This type of statistical analysis should be uniformly done throughout the MS and also extended to Figure 7.

Significance

In my opinion, the most interesting aspect of the MS is the methodology. Based on this, publication is justified and will be of interest to a wider audience. That is why a more detailed analysis of the penetrance of this manipulation across the cell population will be critical.
The application of this method to analyse the composition of the TACC3/Clathrin complex on the spindle is the main biological advance, and the novel information is rather limited but not unimportant.
Overall, if these results can be properly quantified I would recommend publication.

First decision letter

MS ID#: JOCES/2020/255794

MS TITLE: Defining endogenous TACC3-chTOG-clathrin-GTSE1 interactions at the mitotic spindle using induced relocalization

AUTHORS: Ellis L Ryan, James Shelford, Teresa Massam-Wu, Richard Bayliss, and Stephen J Royle
ARTICLE TYPE: Research Article

We have now reached a decision on the above manuscript based on the reviewers reports from review commons. In general the reviewers are positive and largely requested clarifications on a number of issues. Based on your proposed experimental responses to the reviewers questions and concerns I would be happy to see a revised paper and make a quick decision without going back to the reviewers.

We are aware that you may be experiencing disruption to the normal running of your lab that makes experimental revisions challenging.If it would be helpful, we encourage you to contact us to discuss your revision in greater detail. Please send us a point-by-point response indicating where you are able to address concerns raised (either experimentally or by changes to the text) and where you will not be able to do so within the normal timeframe of a revision. We will then provide further guidance. Please also note that we are happy to extend revision timeframes as necessary.

Please ensure that you clearly highlight all changes made in the revised manuscript. Please avoid using 'Tracked changes' in Word files as these are lost in PDF conversion.

I should be grateful if you would also provide a point-by-point response detailing how you have dealt with the points raised by the reviewers in the 'Response to Reviewers' box. Please attend to all of the reviewers' comments. If you do not agree with any of their criticisms or suggestions please explain clearly why this is so.

First revision

Author response to reviewers' comments

We have now completed all of these experiments and address the reviewers’ comments below.
In summary, our revision plan and outcomes:

- Verify effect of chTOG RNAi on GTSE1, clathrin and TACC3 in cells
    - We have done a chTOG knocksideways experiment in normal HeLa using RNAi

© 2021. Published by The Company of Biologists under the terms of the Creative Commons Attribution License (https://creativecommons.org/licenses/by/4.0/).

which confirms our findings with the CRISPR cell line.

- Test new chTOG antibodies
  - Superior antibody identified and used for chTOG quantifications.
- Add more information on the cell lines, including new western blots of GTSE1-FKBP-GFP and details of PCR analyses
  - A new Supplementary Figure S1 shows all PCR and sequencing data for the four cell lines. Western blots of GTSE1-FKBP-GFP were redone (Fig 1B) and a new clone is used throughout the paper.
- Rework the presentation of data in Figures 3 and 4
  - The plots in these figure are enlarged. P-values are added for the live data. Effect sizes for both approaches summarized in Supplementary Table S2.
- Quantify spindle parameters using our existing dataset
  - Analysis completed but no differences observed, at the time points analysed.
- Provide an estimate of knocksideways efficacy
  - Completed and added to Results text.
- Analyze the effect of knocksideways on mitotic progression
  - Data shown in Figure 2C

We thank all three reviewers for their time and their comments on our manuscript.

Reviewer #1 (Evidence, reproducibility and clarity (Required)):

Here Ryan et al. have used localization analysis following induced rapid relocalization of endogenous proteins to investigate the composition and recruitment hierarchy of a clathrin-TACC3-based spindle complex that is important for microtubule organization and stability. The authors generate different HeLa cell lines, each with one of four complex members (TACC3, CLTA, chTOG and GTSE1) endogenously tagged with FKBP-GFP via Cas9- mediated editing. This tag allows rapid recruitment to the mitochondria upon rapamycin addition ("knocksideways"). They ultimately quantify each of the 4 components' localization to the spindle following knocksideways of each component using fluorescently-tagged transfected constructs. The authors' interpretation of the results of this analysis are summarized in the last model figure, in which a core MT-binding complex of clathrin and TACC3 recruit the ancillary components GTSE1 and chTOG. In addition, the authors investigate the contribution of individual clathrin-binding LIDL motifs in GTSE1 to the recruitment of clathrin and GTSE1 to spindles. Their findings here largely agree with and confirm a recent report regarding the contribution of these motifs to GTSE1 recruitment to the spindle. They further analyzed GTSE1 fragments for interphase and mitotic microtubule localization, and identified a second region of GTSE1 required (but not sufficient) for spindle localization. Finally, the authors report that PIK3C2A is not part of this complex, contradicting (correcting) a previously published study.

Major comments:

1. The chTOG-FKBP-GFP cell line the authors generate has only a small fraction of chTOG tagged, and thus should not be used for any conclusions about protein localization dependency on chTOG. Because they were unable to construct a HeLa cell line with all copies tagged, the authors expect that the homozygous knock-in of chTOG-FKBP-GFP is lethal, and thus their experience is appropriate to report. However, the authors should not use this cell line alone to make statements about chTOG dependency. They would have to use similar localization analysis, but after another method to disrupt chTOG (as a second- best approach), such as RNAi. In fact, they have reported this in a previous publication (Booth et al 2011). However, the result was different. There, loss of chTOG resulted in reduced clathrin on spindles, suggesting it may stabilize or help recruit the complex.
Alternatively, they could remove their chTOG data, but this would compromise the "comprehensive" nature of the work.

The referee is correct. The point here is to show the results we had using this approach for all four proteins under study. For this reason, we do not want to remove this data and prefer to show our results “warts-and-all”. We feel that the shortcomings of our approach are honestly presented and discussed in the manuscript. While only a fraction of chTOG was tagged, we should expect some

© 2021. Published by The Company of Biologists under the terms of the Creative Commons Attribution License (https://creativecommons.org/licenses/by/4.0/).

co-removal after its induced mislocalization. Since we saw no change, we concluded that chTOG is auxiliary.
We performed the suggested experiment and found that chTOG RNAi alters the spindle and so quantification is difficult (this was also an issue with the Booth et al 2011 work). However, we have addressed this point experimentally a different way. We have added new data showing knocksideways of transiently expressed chTOG-FKBP-GFP in cells depleted of endogenous chTOG by RNAi. These experiments, summarized in Supplementary Figure S4, verify our findings using the chTOG-FKBP-GFP cell line.

2. The authors initially analyze complex member localization after knocksideways experiments by antibody staining, which has the advantage of analyzing endogenous proteins (versus the later transfected fluorescent constructs). Setting aside potential artefacts from fixation, this would seem to be a better method for controlled analysis to take advantage of their setup (short of generating stable cell lines with second proteins endogenously tagged in a second color - a huge undertaking). The authors conclude that antibody specificity problems confounded their analysis and explained unusual results. However, I think is worth investing a little more effort to sort this out, rather than bringing doubt to the whole data set. Verifying and then using another antibody for chTOG localization would be informative. Of course, the negative control should not be their chTOG- FKBP-GFP line, as it does not relocalize most of chTOG.

In the case of GTSE1, an alternative explanation to antibody specificity issues would be that the GTSE1-FKBP-GFP cell line is not in fact homozygously tagged. Given the low expression levels on the western provided, and the detection of GTSE1 on the spindle in the induced GTSE1-FKBP-GFP cell line (but not TACC3-FKBP-GFP), it seems plausible that an untagged copy remains. If there are multiple copies of GTSE1 in Hela cells, one untagged copy could represent a small fraction of total GTSE1. This should thus be ruled out. GTSE1 clones should be analyzed with more protein extracts loaded - dilutions of the extracts can determine the sensitivity of the blot to lower protein levels. In addition, sequencing of genomic DNA can reveal a small percentage with different reads.

We used a two-pronged approach for assessing relocalization of protein partners (staining vs transfected constructs). The staining approach is superior since endogenous proteins are examined, but it is limited by antibody specificity, background and measuring from different cells. The transfection approach overcomes this limitation but is in turn limited by effects of overexpression and tagging. Together the two approaches allow us, and anyone employing this method, to get a picture of protein complexes. We didn’t want to create the impression that one or other approach is confounded, but the referee is correct that the analysis would benefit from further work.
First, we found an alternative chTOG antibody from Thermo that was able to detect the tagged chTOG protein reliably. Since this antibody was superior, we redid the knocksideways experiments with all four cell lines and stained for chTOG using the Thermo antibody.
Second, we investigated the possibility that an untagged allele of GTSE1 remains. Indeed, loading more of the extracts from this cell line revealed untagged protein and this was then confirmed by PCR. We are very grateful to the Reviewer for challenging us on this. Since we had isolated several potential homozygotes during the original work and have now verified by western blotting and PCR/sequencing that the clone now shown in the paper (A5) is indeed homozygous. We redid all the experiments involving this cell line, which has resulted in new parts to Figures 1BC, 2BC, 3D, 4DF, S3 (current numbering).
Third, PCR verification of all four cell lines has been added as a new Supplementary Figure S1.

3. There is a lot of data contained in the small graphs summarizing quantification of localization in Figs 3 and 4. They would be more accessible to the reader if they were larger and/or an "example" of the chart with labels was present explaining it (essentially what is in the figure legends). Furthermore, there is no statistical test applied to this data that I see.
This is needed. How do authors determine whether there is an "effect"?

Our aim was to compress a lot of information into a small space, while still showing some example primary data. All reviewers raised the same concern which tells us that we went too far towards “data visualization”.
Figures 3 and 4 now have enlarged plots and Figure 4 features an explainer for arrow plots.

© 2021. Published by The Company of Biologists under the terms of the Creative Commons Attribution License (https://creativecommons.org/licenses/by/4.0/).

Minor issues:

1. The GTSE1 constructs used for mutation and localization analysis are 720 amino acids long. A recent study analyzing similar mutations uses a 739 amino acid construct (Rondelet et al 2020). The latter is the predominant transcript in NCBI and Ensembl databases. It appears the construct used by the authors omits the first 19 a.a.. I do not think using the truncated transcript affects conclusions of the manuscript, but it could generate confusion when identifying residues based on a.a.#s of mutant constructs (Fig 6). This should be somehow clarified.

We were aware of the longer transcript but were using the 720 residue form since it is the canonical sequence in Uniprot (https://www.uniprot.org/uniprot/Q9NYZ3). We did not know that the 739 form is the predominant transcript. We agree this is unlikely to affect our work but that the numbering may cause confusion.
We have added a note to the Methods (Molecular Biology section) to accurately describe what we and Rondelet et al. have used.

2. The labeling of constructs in Fig 6C/D is confusing, and appears shifted by eye at places. Please relabel this more clearly.
Corrected.

The recommended new experimental data (Analysis complex member levels on spindles after full perturbation of spindle chTOG; new chTOG antibody stainings in the FKBP lines; reanalysis of GTSE1 DNA/protein in GTSE1-FKBP line) should only require a new antibody/siRNA, plus a few weeks time to repeat the analyses already in the paper with new reagents.

Reviewer #1 (Significance (Required)):

While multiple individual components of this complex have been previously characterized, the structure and nature of the complex formation and its recruitment to microtubules/spindles remains a complex problem that has yet to be solved.

Overall this study represents a comprehensive localization-dependency analysis of the Clathrin-TACC3 based spindle complex using a consistent methodology. Although several of the conclusions of the findings echo previous reports, some of the previous literature is contradictory within itself as well as with the conclusions here. Analyzing all components with a single, rapid-perturbation technique thus has great value to present a clear data set, given that the experimental setup conditions and analysis are solid (a goal to which the majority of comments refer).

Beyond the complex localization/recruitment analysis, two novel findings of this study that emerge are:

a) GTSE1 contains a second, separate protein region, distinct from the clathrin-binding motifs that is required for its localization to the spindle, and most likely a microtubule-interaction site. This suggests that GTSE1 recruitment to the spindle is more complex than previously reported.

b) PI3KC2A, which has been reported previously to be a stabilizing member of this complex, is in fact not a member, nor localizes to spindles, nor displays a mitotic defect after loss. This is important conclusion to be made as it would correct the literature, and avoid future confusion.

Reviewer #2 (Evidence, reproducibility and clarity (Required)):

In this paper, the authors investigate the nature of interactions between members of the TACC3-chTOG-clathrin-GTSE1 complex on the mitotic spindle. By using a series of HeLa cell lines that they have created by CRISPR/Cas9 editing to enable spatial manipulation (knocksideways) of either TACC3, chTOG, clathrin and GTSE1, they show that on spindle microtubules TACC3 and clathrin represent core complex members whereas chTOG and GTSE1 bind to them respectively but not to each other. Additionally, the authors find that the protein PIK3C2A, which has been implicated in this complex previously is in fact not a component of this complex in mitotic cells. The main

© 2021. Published by The Company of Biologists under the terms of the Creative Commons Attribution License (https://creativecommons.org/licenses/by/4.0/).

advance of the paper in my opinion is the endogenous tagging of the proteins for knocksideways experiments since former experiments depended on RNAi silencing and expression of tagged proteins from plasmids, which introduced issues of protein silencing efficiency and plasmid overexpression problems. This approach seems to alleviate these problems, except in the case of chTOG which seems to be lethal in its homozygous variant.

Major comments:

I find the key conclusions regarding the localization of the components of the complex convincing. There are some issues regarding the specificity of antibodies in immunostaining experiments (Fig 3.) and the influence of mCherry-TACC3 expression on distorted localization of the complex prior to knocksideways. However, I think the general conclusion about which complex components (clathrin and TACC3) influence the localization of the other proteins in the complex (chTOG and GTSE1) stands. One thing that I miss from the paper is the data on the consequences on the spindle shape and morphology after knocksideways. I have noticed on images in both Figure 3 and Figure 4 that in some cases distribution of the signal seems to influence quite a bit the spindle morphology. Also, In Figure 3 I have noticed what seems to me a quite big variation in spindle size in tubulin signal in both untreated and rapamycin cells. Since authors have many of these images already, I believe it would be realistic, not costly and of additional value for the paper to provide more data on the consequences of the knocksideways experiments. Change of spindle size, tubulin intensity and DNA/kinetochore misalignment upon knocksideways would be helpful to appreciate more the findings of the paper. More so since the authors on more than one occasion find their motivation in the field of cancer research and spindle stability relation to it. Some data connection to this motivation would be of value.
Experiments seem reproducible.

The focus of the paper is on using the knocksideways methodology to understand a protein complex during mitosis, rather than looking at its function.
Nonetheless, we analyzed several parameters in the dataset that we had: spindle length, width and aspect ratio, and spindle centering measurements. Our analysis did not reveal any major changes in spindle structure or positioning. We have not reported this analysis in the manuscript because the short timeframe of our experiments does not allow us to make a general conclusion here.

Minor comments:

I have some problems with the clarity of Figure 3 and 4. For Figure 3. In Figure 3 plots on the right are a bit small and not easy to read. Some reorganization of the figure might be beneficial. In Figure 4 plots to the right are also too small to be clear. Also, I miss the number of cells (n) I can't see the number of individual arrows because of the size of graphs.

Our aim was to compress a lot of information into a small space, while still showing some example primary data. All reviewers raised the same concern which tells us that we went too far towards “data visualization”.
Figures 3 and 4 now have enlarged plots and Figure 4 features an explainer for arrow plots.

Reviewer #2 (Significance (Required)):

I find that the biggest significance of the paper is in the creation of new tools (cell lines) to study the localization of proteins TACC3, chTOG, clathrin and GTSE1. Cell lines where endogenous proteins can be delocalized rapidly will be of value for scientist working not only in mitosis but such as in the case of clathrin research, vesicle formation and trafficking or p53-dependent apoptosis in the case of GTSE1. In the field of mitosis it will surely help and speed up the research concerning the role of these proteins in spindle assembly and stability.

Field of expertise: mitotic spindle

© 2021. Published by The Company of Biologists under the terms of the Creative Commons Attribution License (https://creativecommons.org/licenses/by/4.0/).

Reviewer #3 (Evidence, reproducibility and clarity (Required)):

Summary:

This papers analyses the chTog/TACC3/clathrin/GTSE1 complex that crosslinks and stabilises microtubule bundles in the mitotic spindle. The authors have developed an elegant knock sideways approach to specifically analyse the effects of removing individual components of the complex from the spindle and study the effect this has on the other interactors. They report, based on these assays that the core of the complex is formed by TACC3 and Clathrin while GTSE1 and chTog are auxiliary interactors. They also refute previous evidence that this complex also incorporates PIK3C2A. Overall, this is an interesting study that distinguishes itself predominantly by its methodology. However, some of the reported results need more thorough analysis to allow convincing conclusions.

Major comments:

1) The knockside way method is the main highlight if this paper. Unlike previous studies by the PI, this time endogenous genes are tagged which is a key advance and allows much better interpretation of the results. I am not sure why the authors have chosen HeLa cells as their model here, given the messed up genome of these cells. A non-transformed cell line would have been preferable, but as a proof of principle study, I think HeLa are acceptable, and I wouldn't expect the authors to repeat all the experiment in another system.
Figure 1,2 and S1 are describing and validating this approach in some detail, but this will require some more work.
The authors state that gene targeting was validated using a combination of PCR, sequencing, Western blotting, but show only the results for westerns. PCR analysis that demonstrates homozygous or heterozygous gene targeting should be shown here.
Another issue is the penetrance of the phenotypes induced by Rapamycin. The authors show nice data of the system working in individual cells but do not give us an idea if this happens in all cells. The localisation of the individual tagged genes should be quantified (ideally with line plots) in 50 randomly chosen mitotic cells with 3 repeats before and after rapamycin treatment. Moreover, the analysis of mitotic duration (Figure S1D) should be extended to include a plus Rapamycin cohort and this should be moved in the main Figure. If the system works only in a small proportion of cells, this should be clearly stated. I don't think this would prevent publication, but it is an important piece of information that is missing.

The Reviewer raises two issues here.
- PCR analysis should be shown. To address this point, we have added details of the PCR and sequencing work done to validate these cell lines to a new Supplementary Figure S1.
- Does knocksideways happen in all cells? The answer to this depends on the transient expression of MitoTrap and sufficient application of rapamycin. We agree that this will be a useful piece of information to add to the manuscript. A related issue is whether knocksideways of complex members affects mitotic progression. We have established through other experiments that rapamycin application to wild-type cells alters mitotic progression, although application of Rapalog does not have this effect. We have 1) quantified efficacy of knocksideways for all four cell lines and this is now reported in the Results section, and 2) analyzed mitotic duration in rapalog-treated cells expressing a rapalog-sensitive MitoTrap. These latter experiments have really enhanced the manuscript since they show that removal of TACC3 or clathrin has a bigger impact on mitosis than removal of GTSE1 or chTOG which is what is expected from our model.

2) Apart from a simple quantification of mitotic duration, I believe a more detailed mitotic phenotype analysis for each knock-side way gene, especially the homozygous targeted clones, should be included. This can involve more high-resolution live cell imaging of mitotic progression with SiR-DNA and GFP-tubulin, using the dark mitotrap.

We did not agree that such an analysis should be included. The focus of this paper is on using the knocksideways methodology to understand a protein complex during mitosis, and not looking at its function. There are several papers on the mitotic phenotypes of these genes probed using RNAi in different cellular systems (examples for chTOG: 10.1101/gad.245603; TACC3/clathrin:

© 2021. Published by The Company of Biologists under the terms of the Creative Commons Attribution License (https://creativecommons.org/licenses/by/4.0/).

10.1038/emboj.2011.15, 10.1242/jcs.075911, 10.1083/jcb.200911091, 10.1083/jcb.200911120; GTSE1: 10.1083/jcb.201606081).
Moreover, our 2013 paper used knocksideways (with RNAi and overexpression) and has a detailed analysis of mitotic progression, microtubule stability, checkpoint activity and kinetochore motions (Cheeseman et al., 2013 doi: 10.1242/jcs.124834).
While a detailed mitotic phenotype analysis is outside the scope of the paper, we nonetheless analyzed several parameters in the datasets we had: spindle length, width and aspect ratio, and spindle centering measurements. Our analysis did not reveal any major changes in spindle structure or positioning. We have not reported this analysis in the manuscript because the short timeframe of our experiments does not allow us to make any general conclusions here.

3) Overall, the quantitative analysis in Figure 3 ,4 and 7 is not good enough and sometimes doesn't fully support the conclusions. In Figure 3,4 a convoluted way of demonstrating the change in localisation is shown and this panel is so small that is almost impossible to read. Also, there is no statistical analysis, and the sample size seems very small . At least 25 cells should be analysed here in 3 repeats. I would suggest to unify the quantification in the MS and use the line plots shown in Figure 5 and 6 and compare each protein before and after rapamycin addition. This is much easier to read and more convincing. The images of the cells panels can be moved to a supplement as they contain very little information. This would generate space to expand the size and depth of the quantitative analysis. Instead of Anova tests, I would recommend using a simple t-test comparing each condition to its relevant control since this is the only relevant comparison in the experiment. Statistical significance should be calculated for each experiment with sufficient sample size. It would also be better to show the individual data points from the three repeats in different colours so that the reproducibility between repeat can be judged.
This type of statistical analysis should be uniformly done throughout the MS and also extended to Figure 7.

Our aim was to compress a lot of information into a small space, while still showing some example primary data. All reviewers raised the same concern which tells us that we went too far towards “data visualization”.
Figures 3 and 4 now have enlarged plots and Figure 4 features an explainer for arrow plots.
Regarding the statistics, we have added p-values to the arrow plots in Figure 4. We have enhanced Table S2 so that it now contains effect sizes and confidence intervals from all of the knocksideways experiments, rather than simple arbitrary definitions.

Reviewer #3 (Significance (Required)):

In my opinion, the most interesting aspect of the MS is the methodology. Based on this, publication is justified and will be of interest to a wider audience. That is why a more detailed analysis of the penetrance of this manipulation across the cell population will be critical.
The application of this method to analyse the composition of the TACC3/Clathrin complex on the spindle is the main biological advance, and the novel information is rather limited but not unimportant.
Overall, if these results can be properly quantified I would recommend publication.

<u>Second decision letter</u>

MS ID#: JOCES/2020/255794

MS TITLE: Defining endogenous TACC3-chTOG-clathrin-GTSE1 interactions at the mitotic spindle using induced relocalization

AUTHORS: Ellis L Ryan, James Shelford, Teresa Massam-Wu, Richard Bayliss, and Stephen J Royle
ARTICLE TYPE: Research Article

© 2021. Published by The Company of Biologists under the terms of the Creative Commons Attribution License (https://creativecommons.org/licenses/by/4.0/).

Thank you for sending your manuscript to Journal of Cell Science through Review Commons. I am happy to tell you that your manuscript has been accepted for publication in Journal of Cell Science, pending standard ethics checks.

© 2021. Published by The Company of Biologists under the terms of the Creative Commons Attribution License (https://creativecommons.org/licenses/by/4.0/).
